# Supplementary material for: Real-time precision opto-control of chemical processes in live cells
Source: Nat Commun. 2022 Jul 27;13:4343. doi: 10.1038/s41467-022-32071-z (PMC9329476; doi:10.1038/s41467-022-32071-z)
Supplement: Supplementary file 1 — Supplementary Information [file 41467_2022_32071_MOESM1_ESM.pdf]

Supplementary information:

Real-time precision opto-control of chemical processes in live cells

Authors

Matthew G. Clark[1], Gil A. Gonzalez[1], Yiyang Luo[1], Jesus A. Aldana-Mendoza[1], Mark S. Carlsen[1], Gregory Eakins[1], Mingji Dai[1,2], and Chi Zhang[1,2,3]*

Affiliations

[1]Department of Chemistry, Purdue University; 560 Oval Dr., West Lafayette, IN 47907, USA.

[2]Purdue Center for Cancer Research; 201 S University St., West Lafayette, IN 47906, USA

[3]Purdue Institute of Inflammation, Immunology and Infectious Disease

*Corresponding author. Email: zhan2017@purdue.edu

Supplementary Note

Supplementary Note 1

In Supplementary Fig. 1a, the optical signal input shown on the left is compared with a voltage threshold that can be set manually using the manual threshold tuning nob or input from the digital threshold input port. The manual & digital switch changes between the threshold selection modes. The opto-control TTL signal is output from the right middle port for AOM control. On the right-hand side, two ports are available to deliver the optical signal output and the digital threshold output for references. The former can be used for image display during RPOC.

Supplementary Note 2

The AOM rise time satisfies

$$\tau_r = 0.65\frac{d}{V}.$$

Here, *d* is the beam diameter, and *V* is the acoustic velocity.

$$d = 2r = 1.22\frac{\lambda}{NA}$$

For the control laser beam at 522 nm and a *NA* value of 0.01, the beam diameter at the AOM crystal is 0.63 x 10^{-4} m. The acoustic velocity (*V*) inside the AOM crystal is 5800 m/s. This gives a ~7 ns AOM rise time for the control laser beam.

Supplementary Note 3

The response time of the RPOC system depends on the optical signals and detection scheme.

For fluorescence detection, the PMT response time is typically 1 ns. The PMT amplifier has a rise time of about 10 ns. Considering the cable length (~ 1 m), the optical path length of the opto-control laser beam (~ 2 m), the AOM rise time of 7 ns, and the 15 ns response time of the comparator circuitry, the response time of the RPOC is ~43 ns.

For SRS signal detection, the rise time of PD is ~ 18 ns. The lock-in time constant is 7 µs. If the pixel dwell time is 10 µs, the RPOC still allows active pixels (APXs) to be activated on the same image pixel. The APX would impact the next pixel. In the oversampling condition used in this work, the two adjacent pixels have a distance of 90 nm. Reducing the lock-in time constant would decrease such an impact.

Supplementary Note 4

An SRS image from MIA PaCa-2 cells was acquired to estimate the spatial resolution of the signal generation (Supplementary Fig. 2). From the Gaussian fitting of a small feature in the image, the spatial resolution is estimated to be ≤ 373 nm. We estimate the TPEF to have a similar spatial resolution as the SRS.

Supplementary Note 5

Spatial overlapping of the excitation and RPOC laser beams is critical to ensure accurate molecular control. To optimize overlapping between the excitation and RPOC laser beams, we used fluorescence microparticles and compared images using both laser beams. Supplementary Figs. 3, a-c illustrate the condition when the beams are not perfectly overlapped and Supplementary Fig. 3s, d-f show the condition with optimized

overlapping. Overlap optimization can be achieved by adjusting two mirrors only in the control laser beam path.

From Supplementary Fig. 3g, we found that the widths of the same particle imaged using TPEF and 522 nm single-photon RPOC laser are 1.04 and 1.35 μm, respectively. The resolution of the TPEF is determined to be ~373 nm from Supplementary Fig. 2. Therefore, the spot size of the RPOC laser 'X' satisfies:

$$1.04 = 0.37 \times 2 + a$$

$$1.35 = 2X + a$$

Here, 'X' is the beam size of the 522 nm RPOC laser, while 'a' is the size of the particles excluding the edges. The solution of these equations gives x=525 nm. This beam size is bigger than the theoretical minimum using a NA=1.2 objective lens, majorly due to the slightly reduced beam size and changing of divergence of the 1st order AOM diffraction, as shown in Fig. 1e.

Supplementary Note 6

As shown in Supplementary Fig. 4a, similar functions to the comparator box 1 with a single threshold selection are available. Besides, a TTL input can be used to perform digital logic calculations with the comparison output from this box. This TTL input can be the TTL output from other comparator boxes. Inverters, an AND gate, and an OR gate are available to achieve different logic combinations.

One function of using two comparator boxes is to select an intensity range for RPOC. The connections of achieving such a function are shown in Supplementary Fig. 5 and Fig. 3a. The upper and lower thresholds are selected by the comparator box 1 and 2, respectively.

The other function of using two comparator boxes is to perform logic calculations from two separate detectors for RPOC. The connections of achieving the AND function are illustrated in Supplementary Fig. 7 and Fig. 3d. Electronic connections for achieving other logic functions are shown in Supplementary Fig. 10.

Supplementary Note 7

In Supplementary Fig. 6, a single-color SRS image, and segmented images highlighting different organelles including LDs, endoplasmic reticulum (ER), nuclei, cytosol, and a composite image are shown. The ER can also be selected from intensity thresholding of SRS images. A single intensity range was used to select APXs on ER in Fig. 3.

Supplementary Note 8

A mixture of fluorescent and nonfluorescent polystyrene (PS) microparticles and nicotinamide adenine dinucleotide hydrogen (NADH) crystals is used to demonstrate the digital AND function for APX determination. As shown in Supplementary Fig. 8a, an SRS image at the PS aromatic stretching band 3060 cm^{-1} as contrast reveals all PS particles in the field of view. In the 450 nm fluorescence channel, both fluorescent PS particles and NADH crystals are visible (Supplementary Fig. 8b). The merging of the two channels highlights only the fluorescent PS particles in yellow (Supplementary Fig. 8c). Using SRS or fluorescence signals, the APXs can be determined for all PS particles or all fluorescence molecules (Supplementary Figs. 8d,e). Using the AND function, APXs are

determined from pixels having signals in both SRS and fluorescence channels, which are the fluorescent PS particles (Supplementary Fig. 8f). Adjusting the V_T from two comparator boxes allows optimization of APXs selected using the AND function. Supplementary Fig. 9 compares APXs from the AND logic using different intensity thresholds selected for two comparator boxes. The optimal condition for selecting APXs in this case is V_{T1}=0.05 V, and V_{T2}=0.125 V.

Supplementary Note 9

General Methods. NMR spectra were recorded on Bruker Avance-III spectrometers (^{1}H at 500 MHz and ^{13}C at 125 MHz). Chemical shifts (δ) were given in ppm with reference to solvent signals [^{1}H NMR: $CHCl_3$ (7.26); ^{13}C NMR: $CDCl_3$ (77.2)]. Column chromatography was performed on silica gel. All reactions sensitive to air or moisture were conducted under argon atmosphere in dry and freshly distilled solvents under anhydrous conditions, unless otherwise noted. Anhydrous tetrahydrofuran (THF) was distilled over sodium benzophenone ketyl under Argon. Anhydrous dimethylformamide (DMF) was distilled over calcium hydride under Argon. All other solvents and reagents were used as obtained from commercial sources without further purification.

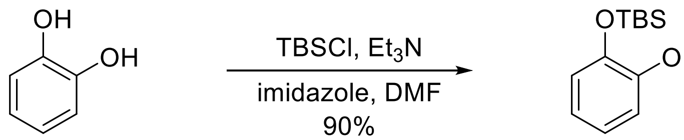

S1

Synthesis of S1. TBSCl (658 mg, 4.4 mmol, 1.0 equiv.) was added to a stirred solution of catechol (580 mg, 5.27 mmol, 1.2 equiv.) and imidazole (850 mg, 11.6 mmol, 2.4 equiv.) in DMF (15 mL), then NEt_3 (1 mL, 7.5 mmol, 1.7 equiv.) was added, and a white precipitate formed. The reaction mixture was stirred overnight and diluted with EtOAc. The combined organic solution was washed with brine, dried over Na_2SO_4, filtered and evaporated. The residue was purified by automatic chromatography (100:0 to 92:8, Hex:EA) to afford product as colorless oil (880 mg, 90%; R_f = 0.60 on 10:1 Hex:EA). ^{1}H NMR matches with literature.[1]

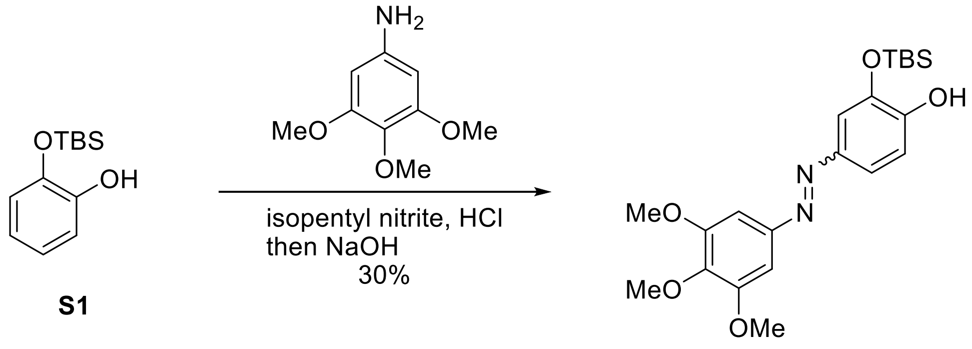

S1 **S2**

Synthesis of S2. To the aniline (536 mg, 2.9 mmol, 1.0 equiv.) were added MeOH (15 mL) and conc. HCl (0.75 mL). The mixture was cooled in an ice bath. A solution of isopentyl nitrite (0.40 mL, 2.9 mmol, 1.02 equiv.) in MeOH (4 mL) was added dropwise and stirred for 30 min in an ice bath. A cold solution of **S1** (656 mg, 2.9 mmol, 1.0 equiv.) in MeOH (6 mL) and NaOH (2.0 M aq., 5.4 mL) was prepared, and added to the mixture dropwise. After stirring in the cold for 30 min, the pH was adjusted to 7 with phosphate buffer, $CHCl_3$ (30 mL) was added, and the aqueous phase was extracted with $CHCl_3$. The combined organic solution was washed with brine, dried over Na_2SO_4, filtered and concentrated. The crude was purified with automatic chromatography (88:12 to 67:33, Hex:EA) to afford product as orange oil (360 mg, 29%, *E*:*Z* = 1:0.72; R_f = 0.31 on 4:1 Hex:EA). ^{1}H NMR matches with literature.[1]

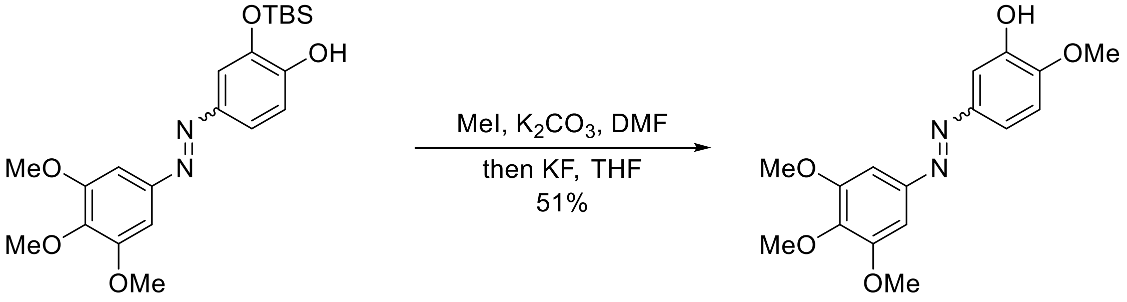

S2 **PST-1**

Synthesis of PST-1. To K_2CO_3 (244 mg, 1.8 mmol, 1.8 equiv.), a solution of **S2** (410 mg, 0.98 mmol, 1.0 euiqv.) in dry DMF (10 mL) and MeI (152 µL, 2.5 mmol, 2.5 equiv.) were added, and the mixture was stirred at room temperature for 2 h. The volatiles were evaporated, then THF (45 mL) and an aqueous solution of KF (1 M, 28 mL) were added to the residue and the mixture stirred at room temperature for 4 h. THF was removed on the rotavap, then water, brine, and phosphate buffer (2.0 M, pH = 7.0, 22 mL) were added and the aqueous phase was extracted with DCM. The combined organic solution was washed with brine and dried over Na_2SO_4, filtered and concentrated. The crude was purified with automactic chromatography in the dark (84:16 to 64:36, Hex:EA) afforded product as an orange solid (160 mg, 51%; R_f = 0.45 on 2:1 Hex:EA). Only one single geometric isomer was isolated. ^{1}H NMR and ^{13}C NMR match with literature.[1]

^{1}H NMR (500 MHz, $CDCl_3$) δ 7.55 (dd, *J* = 8.4, 2.4 Hz, 1H), 7.52 (d, *J* = 2.3 Hz, 1H), 7.22 (s, 2H), 6.98 (d, *J* = 8.4 Hz, 1H), 3.98 (s, 3H), 3.96 (s, 6H), 3.93 (s, 3H). ^{13}C NMR (125 MHz, $CDCl_3$) δ 153.5, 149.2, 148.5, 147.3, 146.2, 140.2, 119.1, 110.1, 106.1, 100.2, 61.0, 56.2, 56.2.

^{1}H and ^{13}C NMR Spectra of PST-1

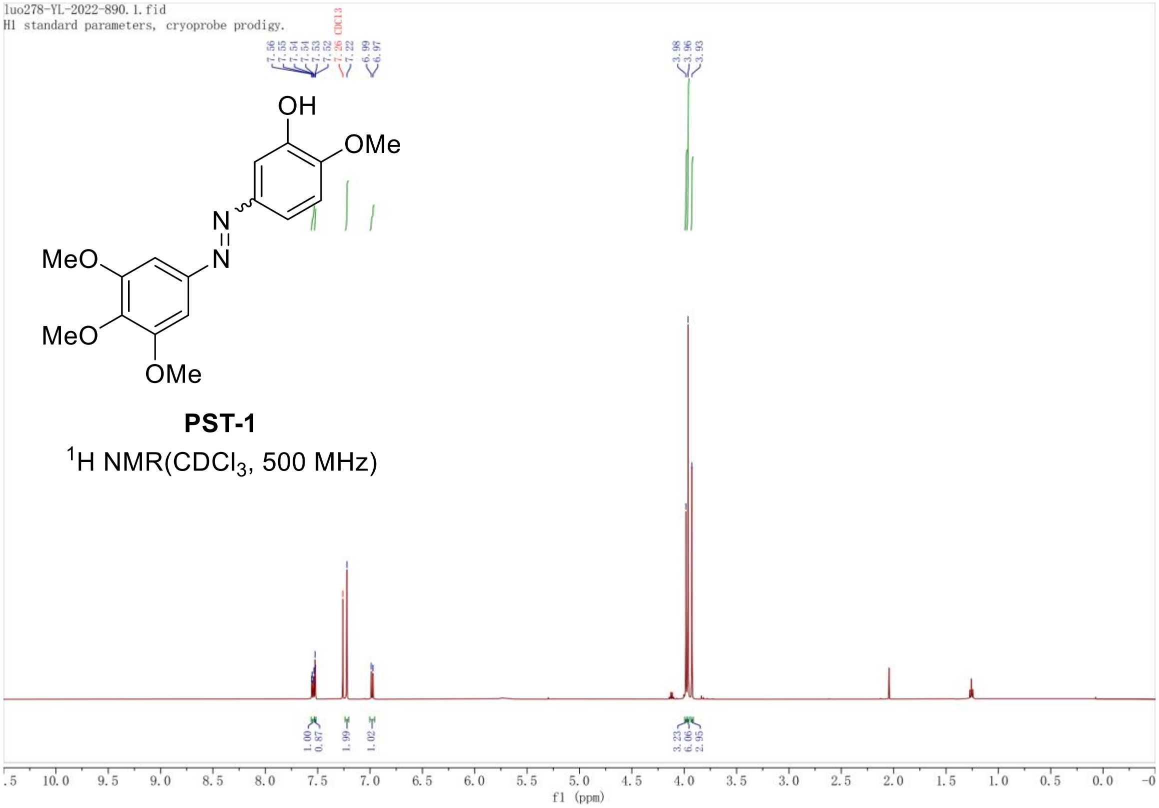

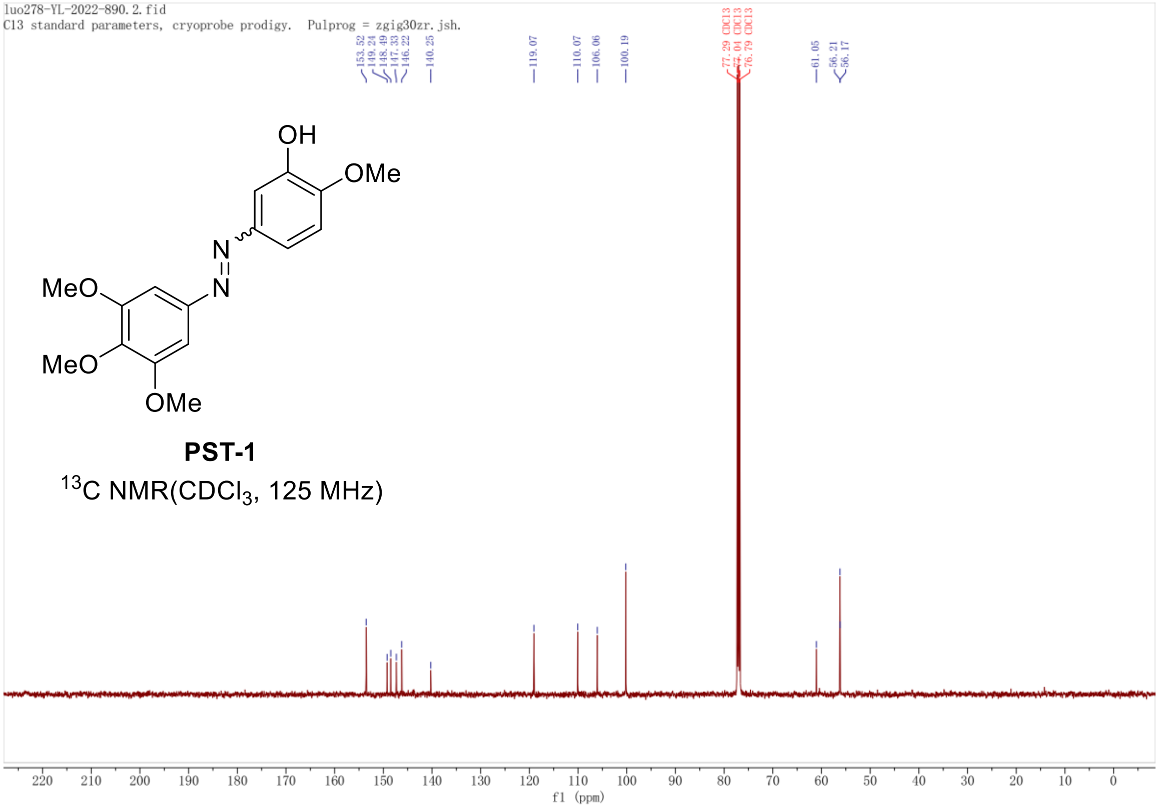

Supplementary Note 10

To quantify the changes in TPEF signals in Fig. 7, we first perform intensity thresholding to select the signal areas in T1, T3, and T4. Then, an AND function in ImageJ is used to quantify the overlapped region of T1 and T3, and T3 and T4, giving areas O13 and O34, respectively. Next, we quantify the TPEF image areas of T1, T3, and T4, and obtain O1, O3, and O4. The percentage of difference is calculated as

A1=(O1-O13)/O1 x 100%

A3=(O3-O13)/O3 x 100%

B3=(O3-O34)/O3 x 100%

B4=(O4-O34)/O4 x 100%

The values of A1, A3, B3, and B4 indicate the TPEF signal differences to the corresponding overlapped areas. A larger value indicates more TPEF signal disparities, which can be correlated to higher EB3 dynamics in cells. For cells treated with PST-1, A1 is before RPOC, and A3, B3, and B4 are after RPOC.

Outlines of the TPEF signals after intensity thresholding and their overlapping images are shown in Supplementary Figs. 13, 15.

Supplementary Figures

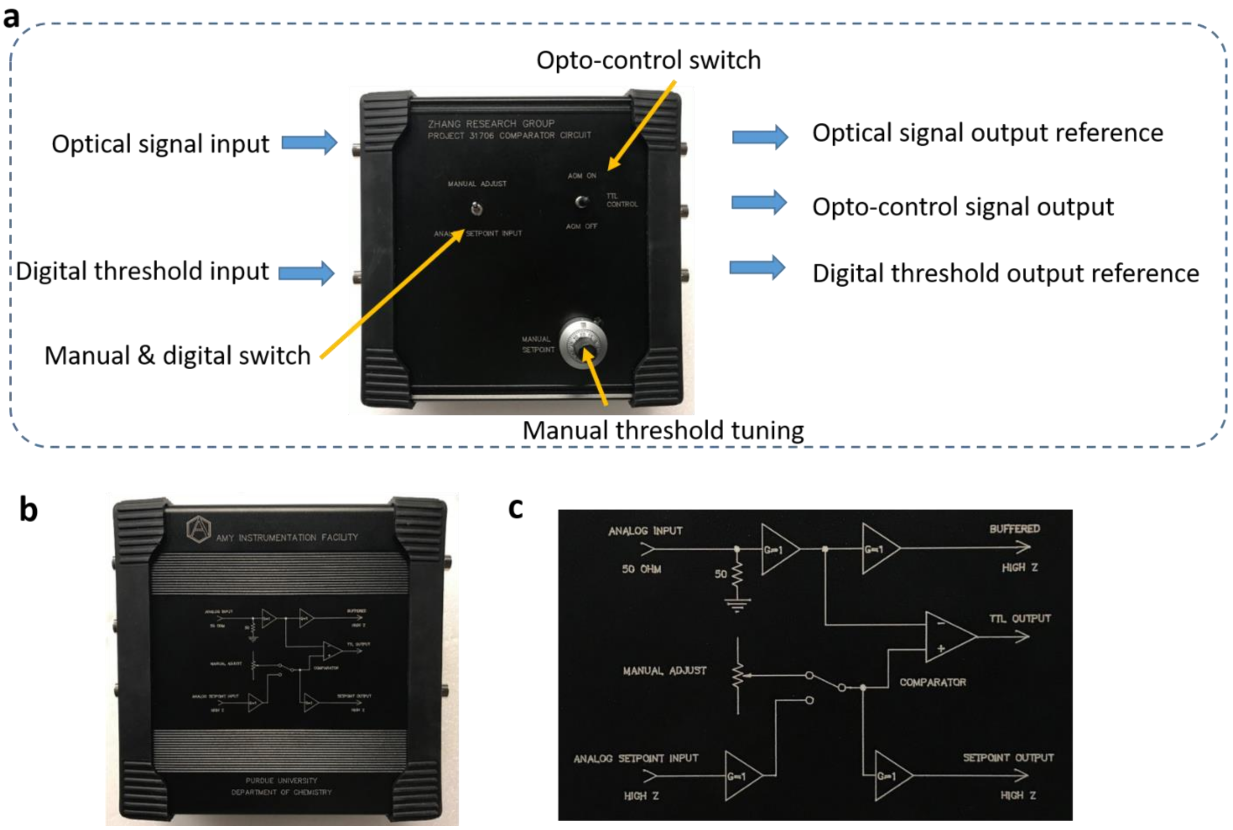

Supplementary Fig. 1. The design of the comparator circuit box 1. a The front of the comparator circuit box with explanations of ports and controls. **b** The back of the comparator circuit box. **c** The electronic configuration of the comparator circuit box.

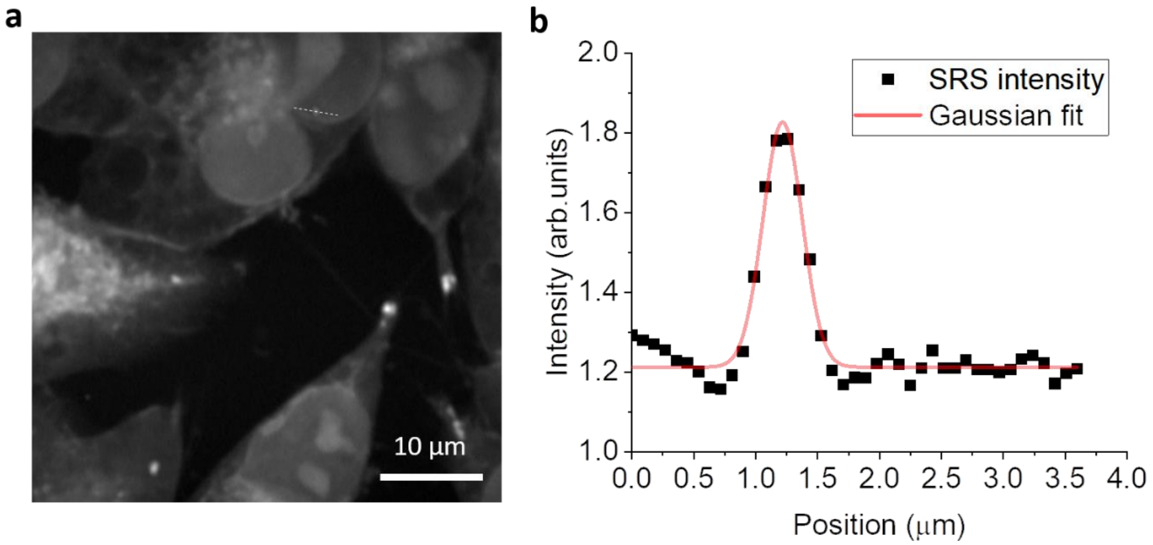

Supplementary Fig. 2. Resolution measurement. a An SRS image of MIA PaCa-2 cells at the 2855 cm^{-1} Raman shift. **b** The SRS intensity profile along the dashed line in panel a. The Gaussian fit quantifies the width of the object, which demonstrates a 373 nm spatial resolution.

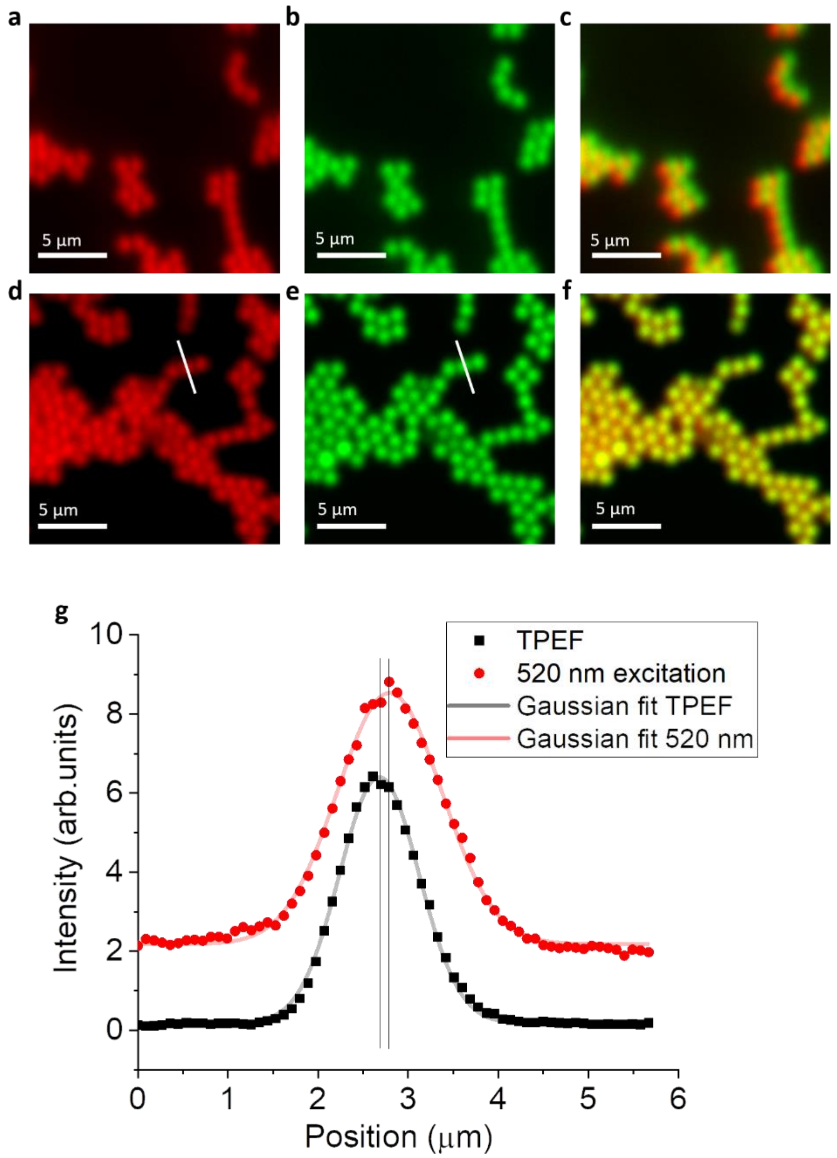

Supplementary Fig. 3. Beam overlapping optimization. a Fluorescence signals from green fluorescent particles excited by the 522 nm RPOC laser beam. **b** TPEF signals from green fluorescent particles excited by the 1045 nm excitation laser beam. **c** Overlay of images in panels A and B, showing an offset due to the misaligned excitation and RPOC laser beams. **d-f** Similar images as shown in panels a-c, after optimization of beam overlapping, showing no image offsets in panel f. **g** Single- and two-photon intensity profiles along the lines in panels d and e. The curves are Gaussian fitting results, showing a peak center offset of ~90 nm.

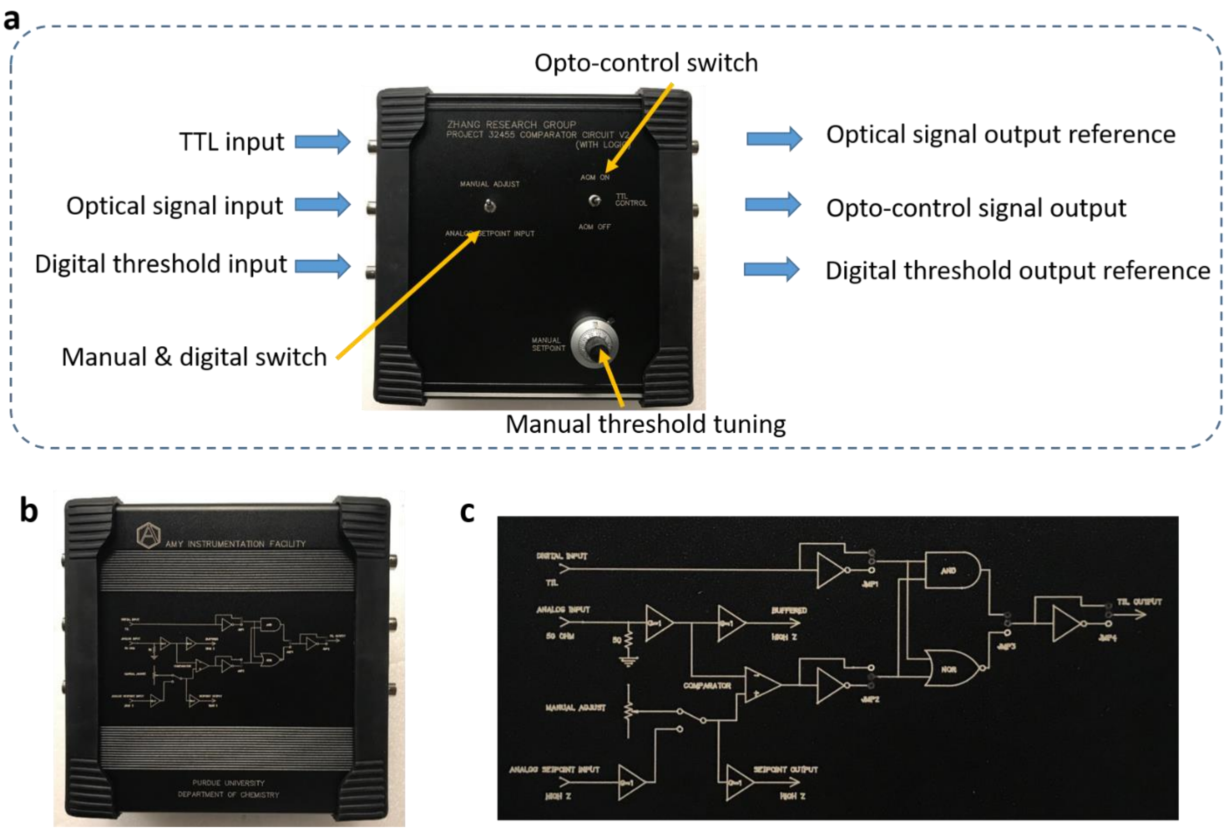

Supplementary Fig. 4. The design of the comparator circuit box 2 with digital logic functions. a The front of the comparator circuit box with explanations of ports and controls. **b** The back of the comparator circuit box. **c** The electronic configuration of the comparator circuit box.

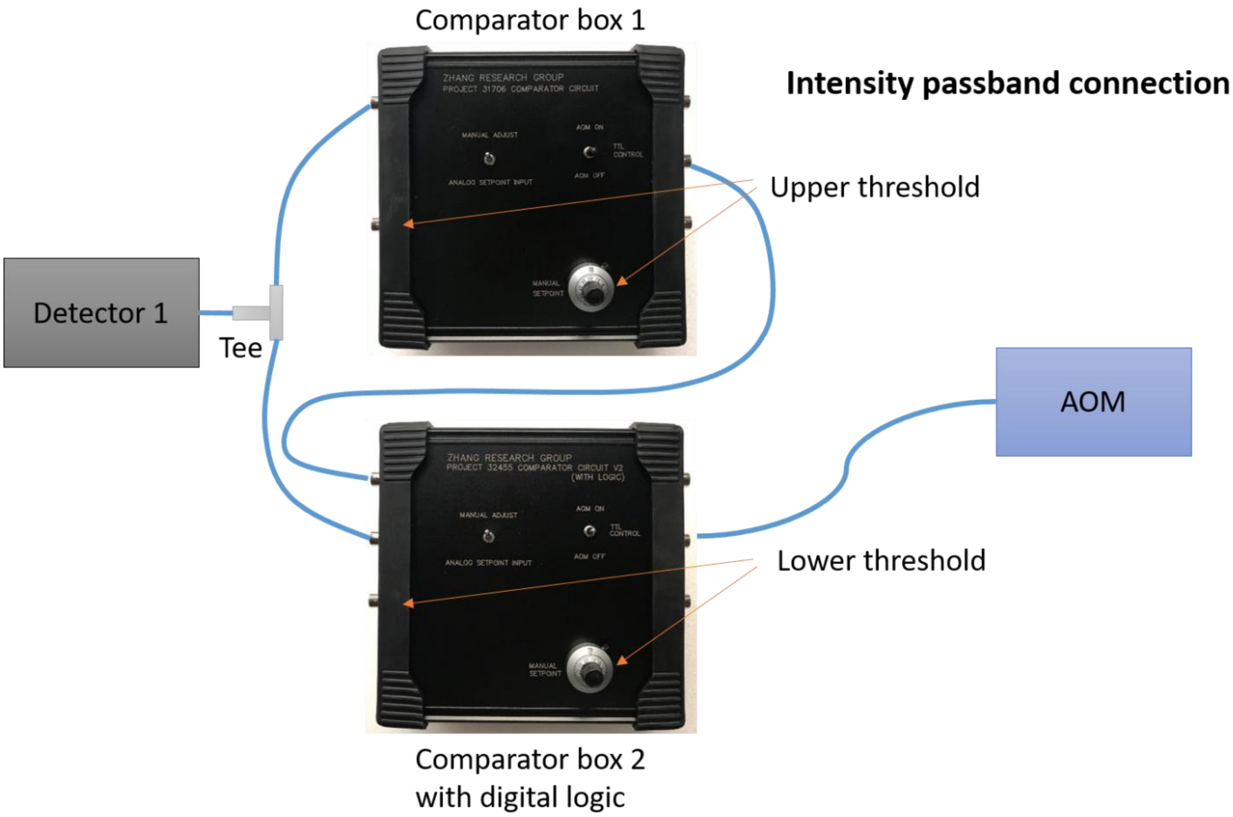

Supplementary Fig. 5. Connections of the two comparator boxes to achieve an intensity passband for APX selection.

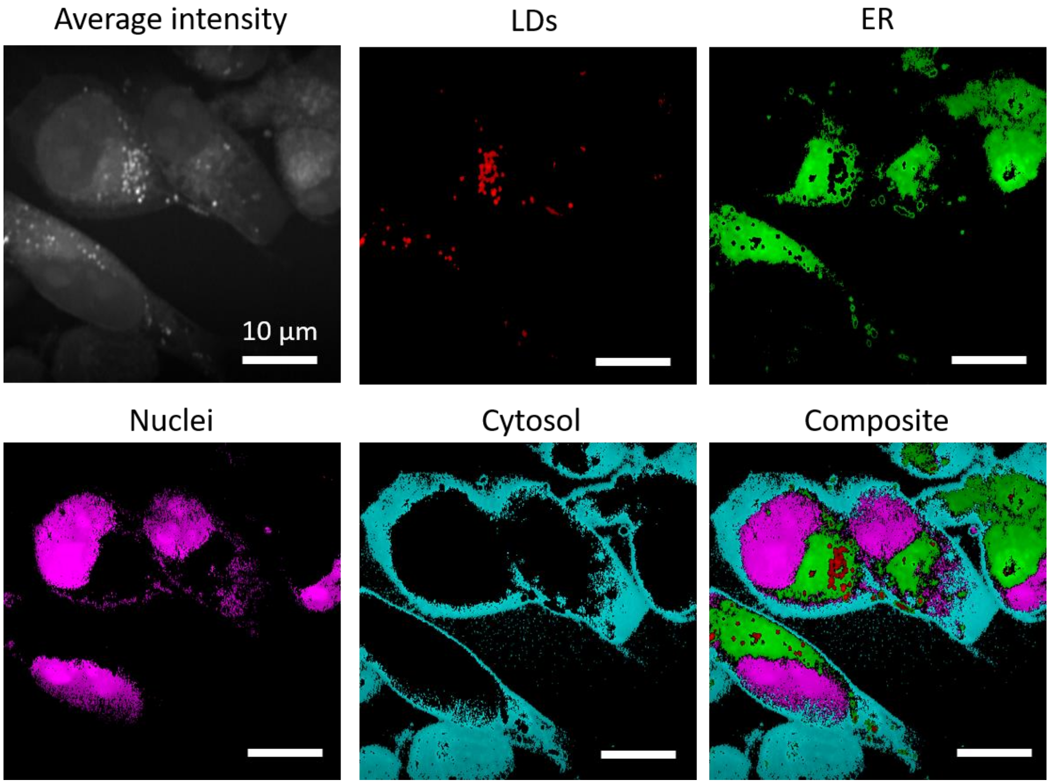

Supplementary Fig. 6. SRS spectral phasor chemical compositions analysis of cells. An SRS image (top left) and the chemical maps showing lipid droplets (LDs), endoplasmic reticulum (ER), nuclei, cytosol, and the composite using four chemical compositions generated by spectral phasor analysis of the hyperspectral SRS images.

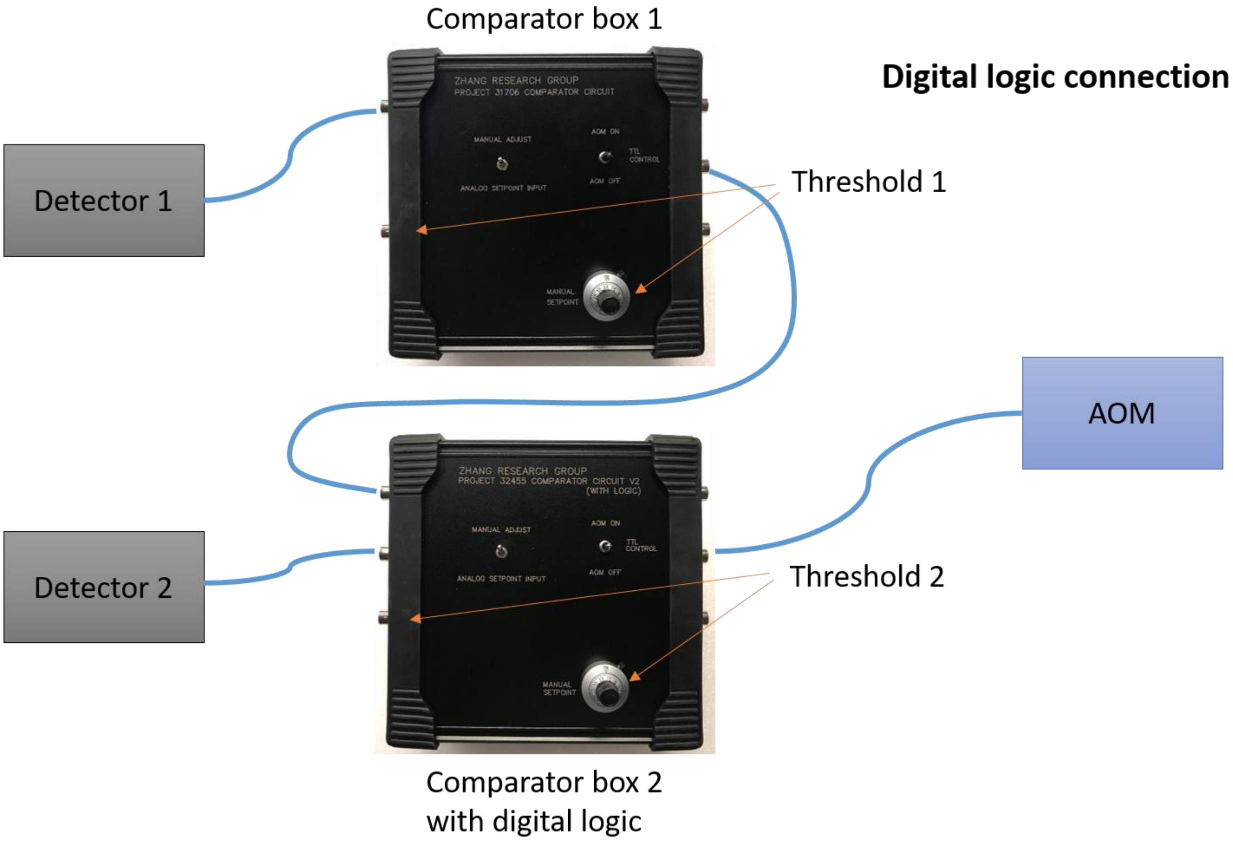

Supplementary Fig. 7. Connections of the two comparator boxes to achieve digital logic functions.

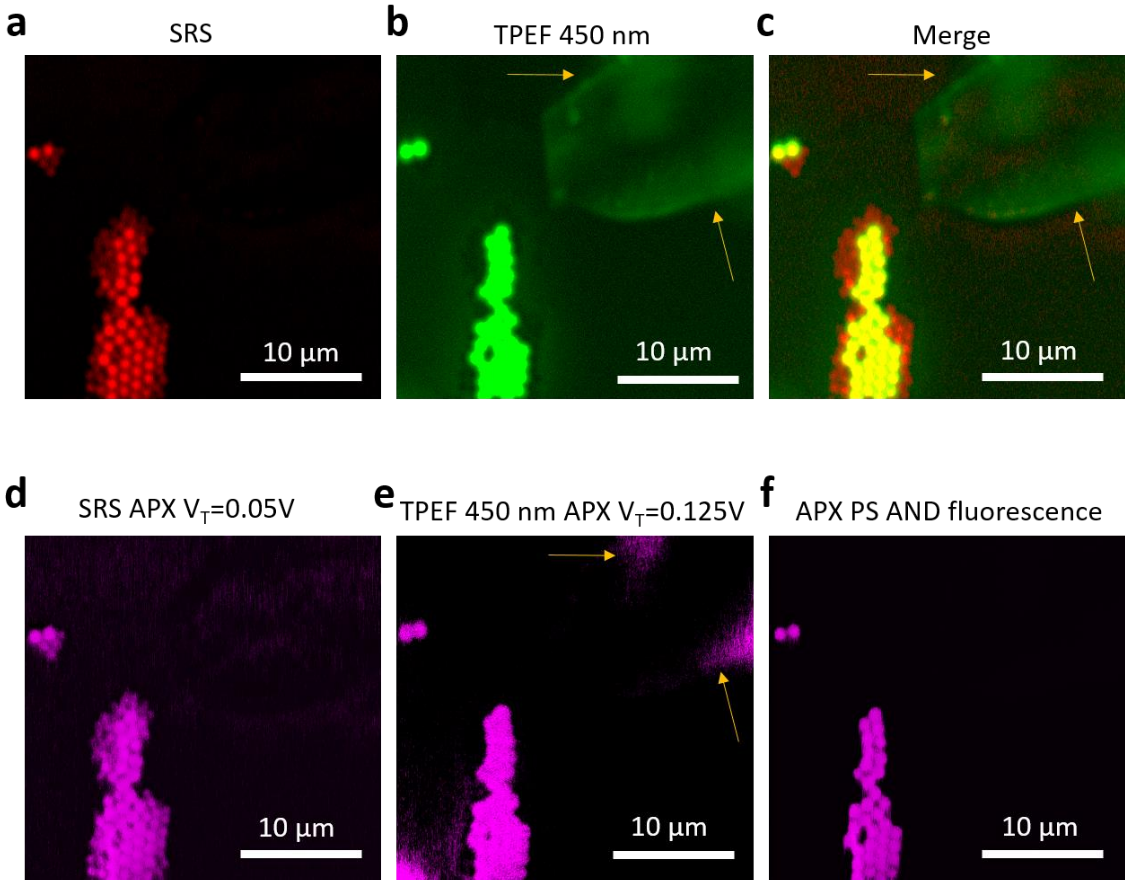

Supplementary Fig. 8. Using the AND function for signals from two detectors. a An SRS image of mixed PS particles and NADH crystals at 3060 cm^{-1} Raman shift. **b** A TPEF image from the 450/106 nm channel of the same field of view of the panel a. **c** Merging the SRS and TPEF images from panels a and b. **d** APXs determined by the SRS signals. **e** APXs determined by the TPEF signals. **f** APXs determined by the SRS AND TPEF pixels.

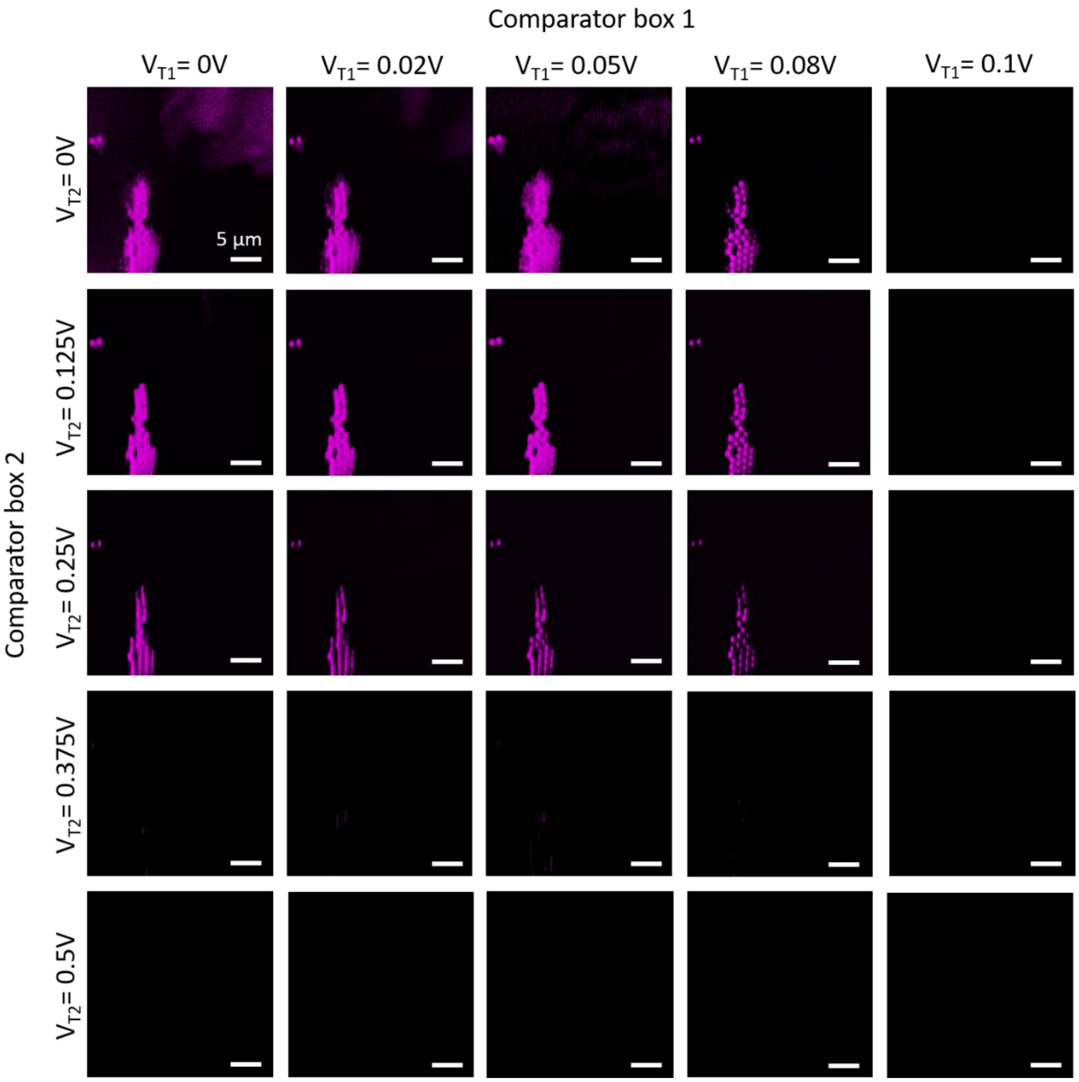

Supplementary Fig. 9. Threshold optimization. Optimizing the voltage threshold values for each comparator box for the AND function. The magenta signals are APXs determined in each condition from the same field of view as in Supplementary Fig. 8.

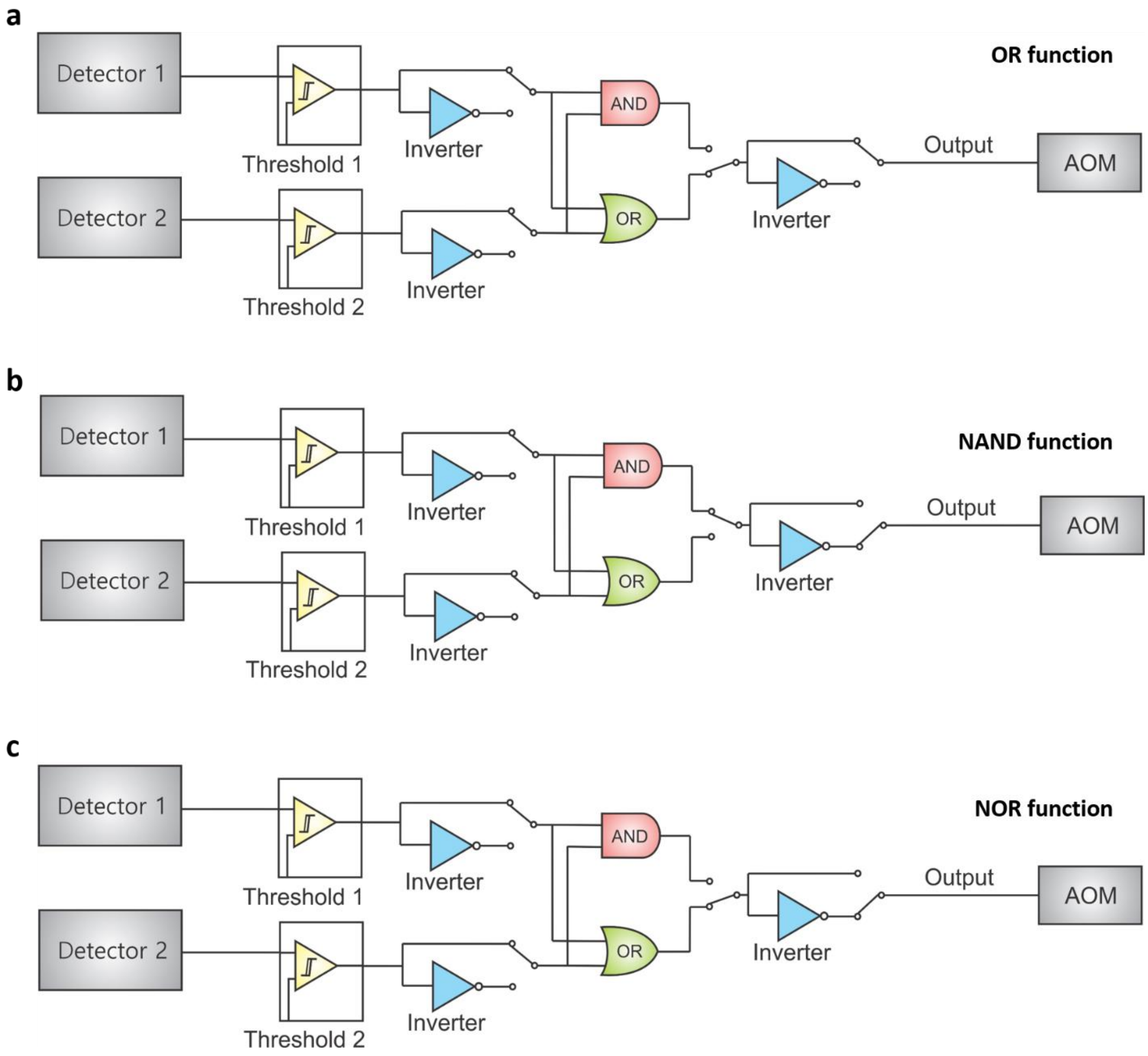

Supplementary Fig. 10. Electronic configurations to achieve the OR, NAND, and NOR logic combinations.

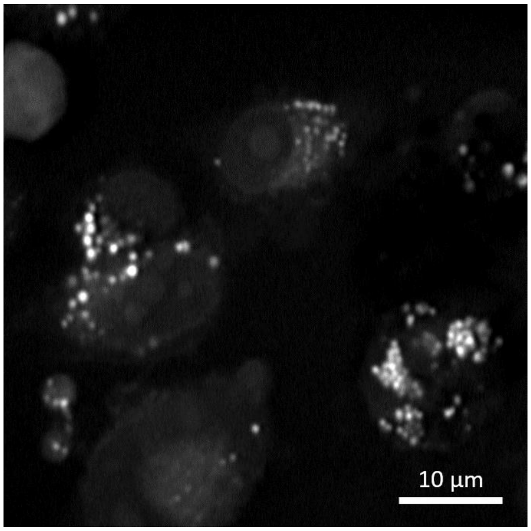

Supplementary Fig. 11. An SRS image of CH_2 stretching signals at 2855 cm^{-1} for images in Fig. 5.

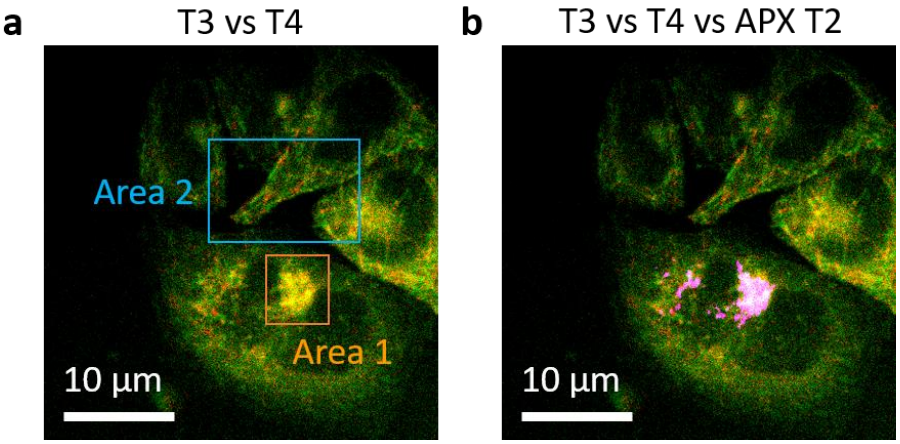

Supplementary Fig. 12. EGFP-EB3 Kyoto HeLa cell TPEF signal analysis. a Overlays of EGFP-EB3 TPEF signals averaged for T3 (green) and T4 (red). **b** An overlay of APX and panel A. Cells are treated with 4 µM PST-1 for 15 min before imaging and RPOC.

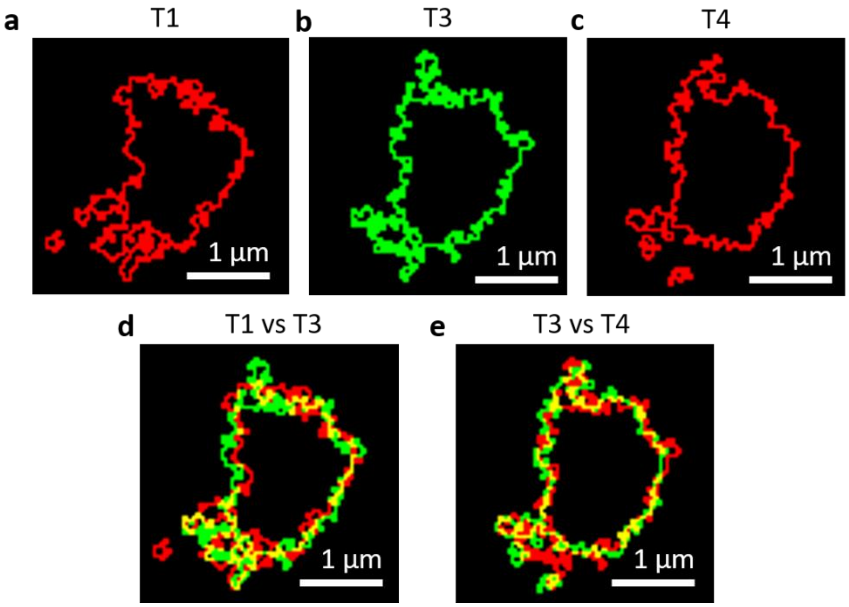

Supplementary Fig. 13. Outlines of centrosome areas before and after RPOC. a-c TPEF intensity outlines of T1, T3, and T4 in the centrosome area, respectively. **d,e** TPEF intensity outline overlap of T1 vs T3, and T3 vs T4, respectively. Cells are treated with 4 µM PST-1 for 15 min before imaging and RPOC.

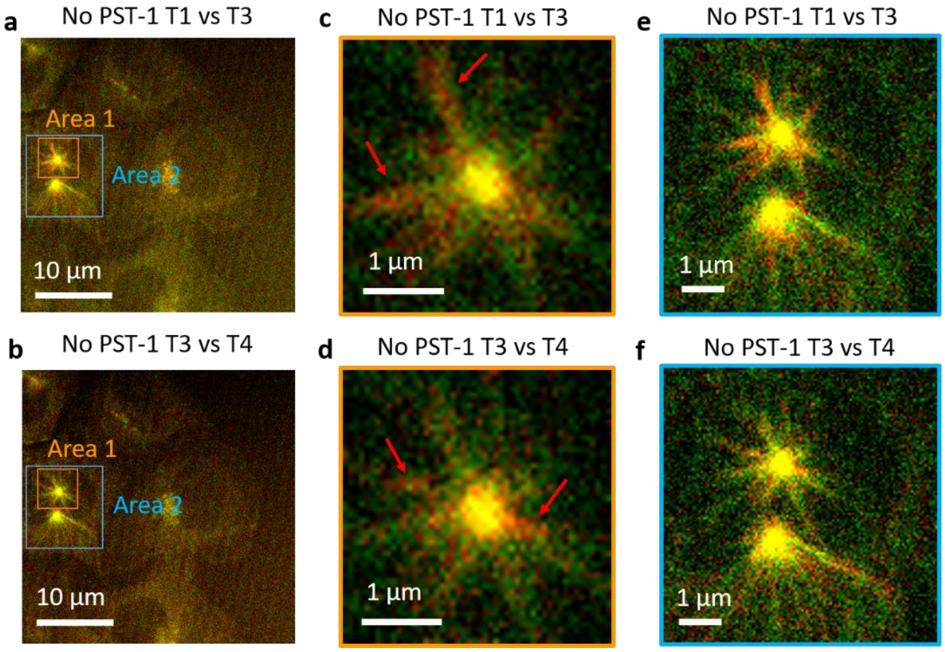

Supplementary Fig. 14. EGFP-EB3 HeLa cell TPEF signal analysis for untreated cells. a,b Overlays of EGFP-EB3 TPEF signals averaged for T1 (red) and T3 (green), and T3 (green) and T4 (red), respectively. Cells are not treated with PST-1. **c,d** Area 1 around the centrosome in panels a and b. **e,f** Area 2 around the centrosome in panels a and b.

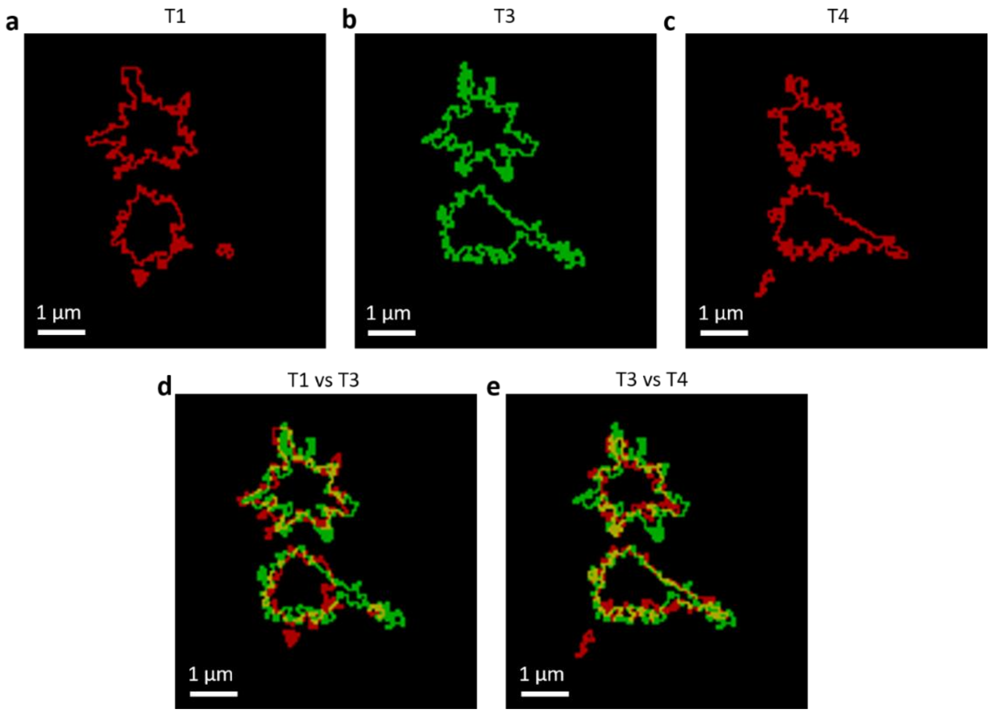

Supplementary Fig. 15. Outlines of centrosome areas for untreated EGFP-EB3 HeLa cells. a-c TPEF intensity outline of T1, T3, and T4 in the centrosome area, respectively. **d,e** TPEF intensity outline overlap of T1 vs T3, and T3 vs T4, respectively. Cells are not treated with PST-1.

Supplementary References

1 Borowiak, M. *et al.* Photoswitchable inhibitors of microtubule dynamics optically control mitosis and cell death. *Cell* **162**, 403-411 (2015).
