# Supplementary material for: Real-time precision opto-control of chemical processes in live cells
Source: Nat Commun. 2022 Jul 27;13:4343. doi: 10.1038/s41467-022-32071-z (PMC9329476; doi:10.1038/s41467-022-32071-z)
Supplement: Supplementary file 2 — Description of Additional Supplementary Files [file 41467_2022_32071_MOESM2_ESM.pdf]

Title: Supplementary Movie 1.
Description: APXs determination by SRS signals from mixed polymer microparticles when scanning the Raman shift from 2800 cm^{-1} to 3100 cm^{-1}.

Title: Supplementary Movie 2.
Description: Time-lapse SRS images (left) and APXs (right) in live MIA PaCa-2 cells at 2.2 s per frame.

Title: Supplementary Movie 3.
Description: Time-lapse SRS images (left) and APXs (right) of a single lipid droplet in a live MIA PaCa-2 cell at 2.2 s per frame.

Title: Supplementary Movie 4.
Description: An illustration of axial scanning for APX determination. Grey: SRS signals at 2855 cm^{-1}; Magenta: APXs.

Title: Supplementary Movie 5.
Description: An illustration of APXs in a 3D volume. Grey: SRS signals at 2855 cm^{-1}; Magenta: APXs.

Title: Supplementary Movie 6.
Description: Comparison between T1 vs T3, and T3 vs T4 for Area 1 (associated with APXs) in Figs. 7g, h.

Title: Supplementary Movie 7.
Description: Comparison between T1 vs T3, and T3 vs T4 for Area 2 (not associated with APXs) Figs. 7k, l.

Title: Supplementary Movie 8.
Description: Comparison between T1 vs T3, and T3 vs T4 for the areas selected in Supplementary Figs. 14c, d.

Title: Supplementary Movie 9.
Description: Comparison between T1 vs T3, and T3 vs T4 for larger areas in Supplementary Figs. 14e, f.

Title: Supplementary Movie 10.
Description: LD dynamics for Fig 8. a-f for Kyoto HeLa cells before and during RPOC. APXs are shown as magenta.

Title: Supplementary Movie 11.
Description: LD dynamics for Kyoto HeLa cells before, during, and after RPOC. APXs are shown as magenta.
