# Supplementary material for: Real-time precision opto-control of chemical processes in live cells
Source: Nat Commun. 2022 Jul 27;13:4343. doi: 10.1038/s41467-022-32071-z (PMC9329476; doi:10.1038/s41467-022-32071-z)
Supplement: Supplementary file 15 — Reporting Summary [file 41467_2022_32071_MOESM15_ESM.pdf]

nature portfolio

Corresponding author(s): Chi Zhang

Last updated by author(s): 06-27-2022

Reporting Summary

Nature Portfolio wishes to improve the reproducibility of the work that we publish. This form provides structure for consistency and transparency in reporting. For further information on Nature Portfolio policies, see our Editorial Policies and the Editorial Policy Checklist.

Statistics

For all statistical analyses, confirm that the following items are present in the figure legend, table legend, main text, or Methods section.

n/a	Confirmed	
☐	☒	The exact sample size (n) for each experimental group/condition, given as a discrete number and unit of measurement
☐	☒	A statement on whether measurements were taken from distinct samples or whether the same sample was measured repeatedly
☒	☐	The statistical test(s) used AND whether they are one- or two-sided<br>*Only common tests should be described solely by name; describe more complex techniques in the Methods section.*
☒	☐	A description of all covariates tested
☐	☒	A description of any assumptions or corrections, such as tests of normality and adjustment for multiple comparisons
☐	☒	A full description of the statistical parameters including central tendency (e.g. means) or other basic estimates (e.g. regression coefficient) AND variation (e.g. standard deviation) or associated estimates of uncertainty (e.g. confidence intervals)
☒	☐	For null hypothesis testing, the test statistic (e.g. F, t, r) with confidence intervals, effect sizes, degrees of freedom and P value noted<br>*Give P values as exact values whenever suitable.*
☒	☐	For Bayesian analysis, information on the choice of priors and Markov chain Monte Carlo settings
☒	☐	For hierarchical and complex designs, identification of the appropriate level for tests and full reporting of outcomes
☒	☐	Estimates of effect sizes (e.g. Cohen's d, Pearson's r), indicating how they were calculated

Our web collection on statistics for biologists contains articles on many of the points above.

Software and code

Policy information about availability of computer code

Data collection	Lab-written software based on LabVIEW 2020 was used for image acqusition,<br>Rayci 64bit was used to acquire beam intensity profiles
Data analysis	ImageJ 1.52a was used for image analysis and display,<br>Origin 2021 was used to plotting curves and fitting,<br>MATLAB R2021b was used in lipid droplet trajectory analysis

For manuscripts utilizing custom algorithms or software that are central to the research but not yet described in published literature, software must be made available to editors and reviewers. We strongly encourage code deposition in a community repository (e.g. GitHub). See the Nature Portfolio guidelines for submitting code & software for further information.

Data

Policy information about availability of data

All manuscripts must include a data availability statement. This statement should provide the following information, where applicable:

- Accession codes, unique identifiers, or web links for publicly available datasets
- A description of any restrictions on data availability
- For clinical datasets or third party data, please ensure that the statement adheres to our policy

All data, code, and materials used in the analysis are available in the corresponding authors' lab. Source data are provided with this paper. The image and analysis

data generated in this study have been deposited in the figshare database. [https://figshare.com/s/c19c9929603955ad62c7].

Human research participants

Policy information about studies involving human research participants and Sex and Gender in Research.

Reporting on sex and gender	N.A.
Population characteristics	N.A.
Recruitment	N.A.
Ethics oversight	N.A.

Note that full information on the approval of the study protocol must also be provided in the manuscript.

Field-specific reporting

Please select the one below that is the best fit for your research. If you are not sure, read the appropriate sections before making your selection.

[x] Life sciences [ ] Behavioural & social sciences [ ] Ecological, evolutionary & environmental sciences

For a reference copy of the document with all sections, see nature.com/documents/nr-reporting-summary-flat.pdf

Life sciences study design

All studies must disclose on these points even when the disclosure is negative.

Sample size	Sample size was determined based on similar studies in this field.
Data exclusions	No data exlusions
Replication	Images in Figs. 2-6 were repeated at least 3 times with similar results.<br>Images in Fig 5 were repeated twice with similar results.<br>Images in Figs 8a-i were repeated 8 times to generate the statistical data in Fig. 8j.<br>Images in the supplementary figures were repeated at least once.
Randomization	Image field-of-views in the sample dish are randomly selected
Blinding	The authors are not blinded to the group allocation and analysis

Reporting for specific materials, systems and methods

We require information from authors about some types of materials, experimental systems and methods used in many studies. Here, indicate whether each material, system or method listed is relevant to your study. If you are not sure if a list item applies to your research, read the appropriate section before selecting a response.

Materials & experimental systems

n/a	Involved in the study	
[x]	[ ]	Antibodies
[ ]	[x]	Eukaryotic cell lines
[x]	[ ]	Palaeontology and archaeology
[x]	[ ]	Animals and other organisms
[x]	[ ]	Clinical data
[x]	[ ]	Dual use research of concern

Methods

n/a	Involved in the study	
[x]	[ ]	ChIP-seq
[x]	[ ]	Flow cytometry
[x]	[ ]	MRI-based neuroimaging

Eukaryotic cell lines

Policy information about cell lines and Sex and Gender in Research

Cell line source(s)	MIA PaCa2 cell line was obtained from ATCC<br>EB3-EGFP HeLa cells were purchased from BIoHippo
Authentication	MIA PaCa2 cell line was not authenticated; EB3-EGFP signals are visualized using fluorescence microscopy for EB3-EGFP HeLa cells
Mycoplasma contamination	Not tested
Commonly misidentified lines<br>(See ICLAC register)	No commonly misidentified lines are used
