# Supplementary figures and images for: Real-time precision opto-control of chemical processes in live cells (part 2 of 2)
Source: Nat Commun. 2022 Jul 27;13:4343. doi: 10.1038/s41467-022-32071-z (PMC9329476; doi:10.1038/s41467-022-32071-z)

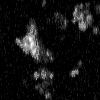

Supplement: Supplementary file 16 — Source Data [file 41467_2022_32071_MOESM16_ESM.zip › Figure 5/Mia PaCa 2 cells 30 mW 80 mW 0.003 40x two box U0.12 L0.1 active pixel 01 frame 2 cropped.png]

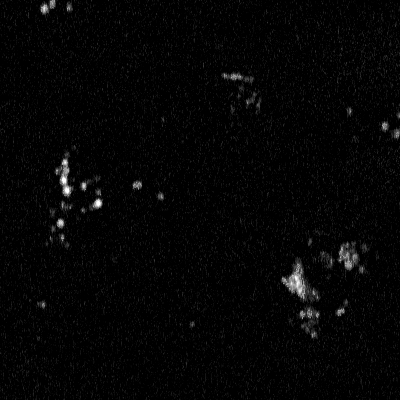

Supplement: Supplementary file 16 — Source Data [file 41467_2022_32071_MOESM16_ESM.zip › Figure 5/Mia PaCa 2 cells 30 mW 80 mW 0.003 40x two box U0.12 L0.1 active pixel 01 frame 2.png]

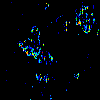

Supplement: Supplementary file 16 — Source Data [file 41467_2022_32071_MOESM16_ESM.zip › Figure 5/Mia PaCa 2 cells 30 mW 80 mW 0.003 40x two box U0.12 L0.1 active pixel 01 intensity difference cropped.png]

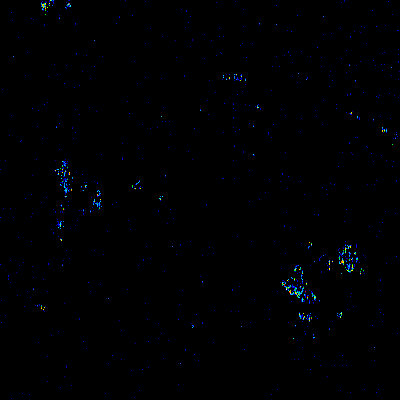

Supplement: Supplementary file 16 — Source Data [file 41467_2022_32071_MOESM16_ESM.zip › Figure 5/Mia PaCa 2 cells 30 mW 80 mW 0.003 40x two box U0.12 L0.1 active pixel 01 intensity difference.png]

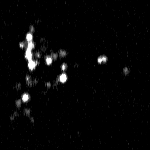

Supplement: Supplementary file 16 — Source Data [file 41467_2022_32071_MOESM16_ESM.zip › Figure 5/Mia PaCa 2 cells 30 mW 80 mW 0.003 40x two box U0.4 L0.15 active pixel 01 frame 1 #2 cropped.png]

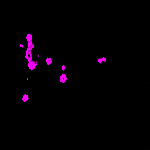

Supplement: Supplementary file 16 — Source Data [file 41467_2022_32071_MOESM16_ESM.zip › Figure 5/Mia PaCa 2 cells 30 mW 80 mW 0.003 40x two box U0.4 L0.15 active pixel 01 frame 1 Active pixels #2 cropped.png]

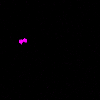

Supplement: Supplementary file 16 — Source Data [file 41467_2022_32071_MOESM16_ESM.zip › Figure 5/Mia PaCa 2 cells 30 mW 80 mW 0.003 40x two box U0.4 L0.15 active pixel 01 frame 1 Active pixels cropped.png]

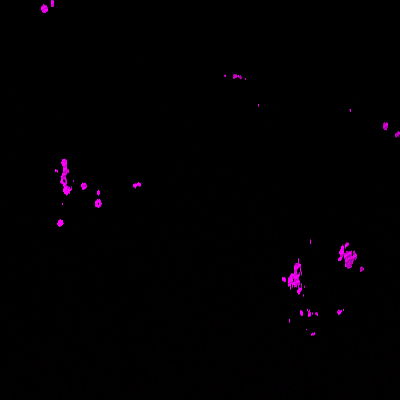

Supplement: Supplementary file 16 — Source Data [file 41467_2022_32071_MOESM16_ESM.zip › Figure 5/Mia PaCa 2 cells 30 mW 80 mW 0.003 40x two box U0.4 L0.15 active pixel 01 frame 1 Active pixels.png]

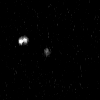

Supplement: Supplementary file 16 — Source Data [file 41467_2022_32071_MOESM16_ESM.zip › Figure 5/Mia PaCa 2 cells 30 mW 80 mW 0.003 40x two box U0.4 L0.15 active pixel 01 frame 1 cropped.png]

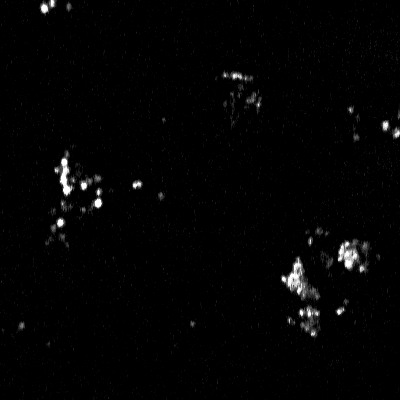

Supplement: Supplementary file 16 — Source Data [file 41467_2022_32071_MOESM16_ESM.zip › Figure 5/Mia PaCa 2 cells 30 mW 80 mW 0.003 40x two box U0.4 L0.15 active pixel 01 frame 1.png]

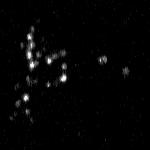

Supplement: Supplementary file 16 — Source Data [file 41467_2022_32071_MOESM16_ESM.zip › Figure 5/Mia PaCa 2 cells 30 mW 80 mW 0.003 40x two box U0.4 L0.15 active pixel 01 frame 2 #2 cropped.png]

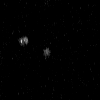

Supplement: Supplementary file 16 — Source Data [file 41467_2022_32071_MOESM16_ESM.zip › Figure 5/Mia PaCa 2 cells 30 mW 80 mW 0.003 40x two box U0.4 L0.15 active pixel 01 frame 2 cropped.png]

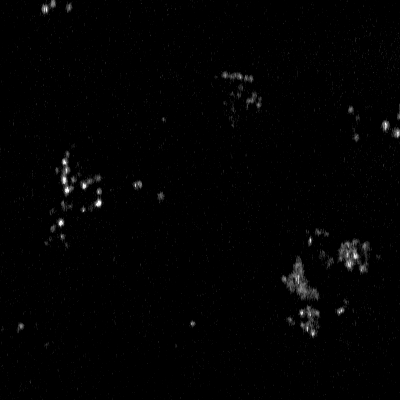

Supplement: Supplementary file 16 — Source Data [file 41467_2022_32071_MOESM16_ESM.zip › Figure 5/Mia PaCa 2 cells 30 mW 80 mW 0.003 40x two box U0.4 L0.15 active pixel 01 frame 2.png]

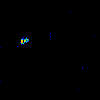

Supplement: Supplementary file 16 — Source Data [file 41467_2022_32071_MOESM16_ESM.zip › Figure 5/Mia PaCa 2 cells 30 mW 80 mW 0.003 40x two box U0.4 L0.15 active pixel 01 intensity difference cropped.png]

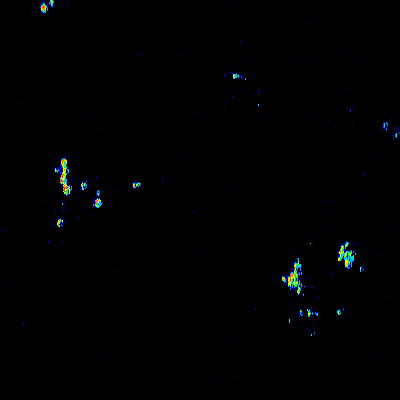

Supplement: Supplementary file 16 — Source Data [file 41467_2022_32071_MOESM16_ESM.zip › Figure 5/Mia PaCa 2 cells 30 mW 80 mW 0.003 40x two box U0.4 L0.15 active pixel 01 intensity difference.png]

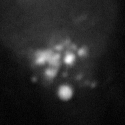

Supplement: Supplementary file 16 — Source Data [file 41467_2022_32071_MOESM16_ESM.zip › Figure 6/CH crop.png]

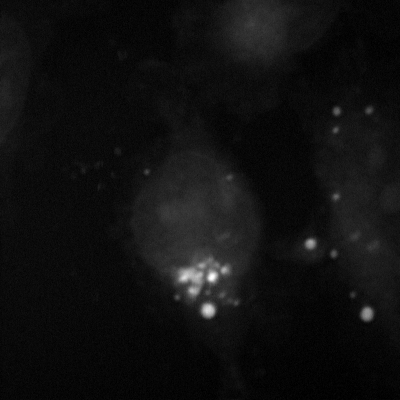

Supplement: Supplementary file 16 — Source Data [file 41467_2022_32071_MOESM16_ESM.zip › Figure 6/CH.png]

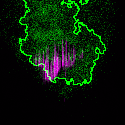

Supplement: Supplementary file 16 — Source Data [file 41467_2022_32071_MOESM16_ESM.zip › Figure 6/ER AP crop.png]

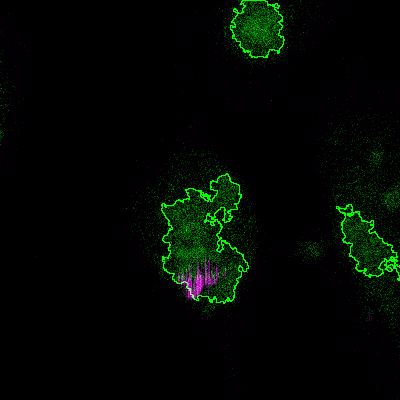

Supplement: Supplementary file 16 — Source Data [file 41467_2022_32071_MOESM16_ESM.zip › Figure 6/ER AP.png]

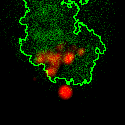

Supplement: Supplementary file 16 — Source Data [file 41467_2022_32071_MOESM16_ESM.zip › Figure 6/ER CMTE Crop.png]

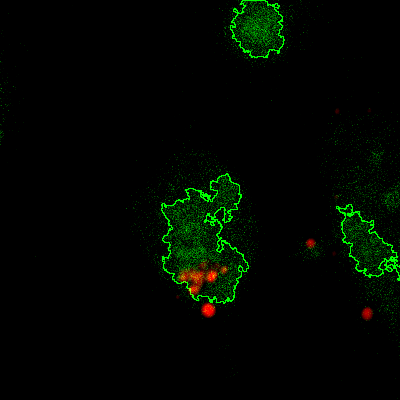

Supplement: Supplementary file 16 — Source Data [file 41467_2022_32071_MOESM16_ESM.zip › Figure 6/ER CMTE.png]

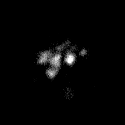

Supplement: Supplementary file 16 — Source Data [file 41467_2022_32071_MOESM16_ESM.zip › Figure 6/Subtraction crop.png]

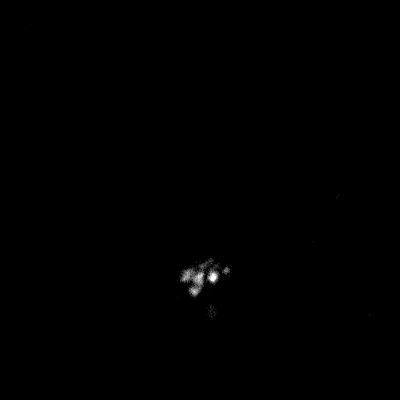

Supplement: Supplementary file 16 — Source Data [file 41467_2022_32071_MOESM16_ESM.zip › Figure 6/Subtraction.png]

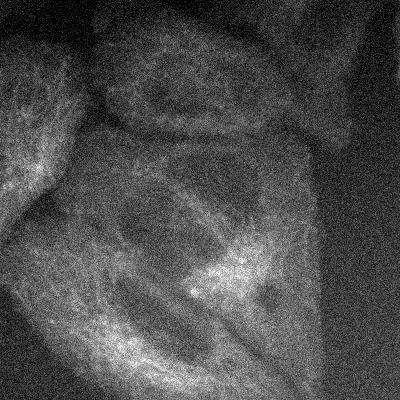

Supplement: Supplementary file 16 — Source Data [file 41467_2022_32071_MOESM16_ESM.zip › Figure 7/After blue.png]

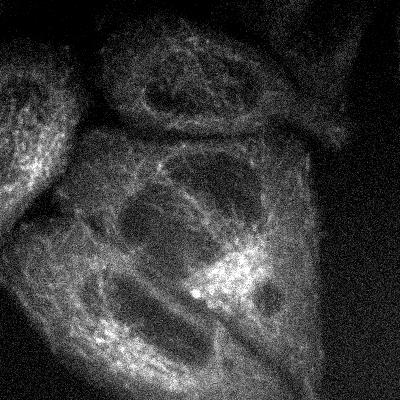

Supplement: Supplementary file 16 — Source Data [file 41467_2022_32071_MOESM16_ESM.zip › Figure 7/Before blue.png]

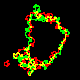

Supplement: Supplementary file 16 — Source Data [file 41467_2022_32071_MOESM16_ESM.zip › Figure 7/Outline T1 vs T3.png]

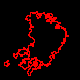

Supplement: Supplementary file 16 — Source Data [file 41467_2022_32071_MOESM16_ESM.zip › Figure 7/Outline T1.png]

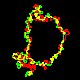

Supplement: Supplementary file 16 — Source Data [file 41467_2022_32071_MOESM16_ESM.zip › Figure 7/Outline T3 vs T4.png]

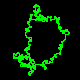

Supplement: Supplementary file 16 — Source Data [file 41467_2022_32071_MOESM16_ESM.zip › Figure 7/Outline T3.png]

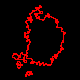

Supplement: Supplementary file 16 — Source Data [file 41467_2022_32071_MOESM16_ESM.zip › Figure 7/Outline T4.png]

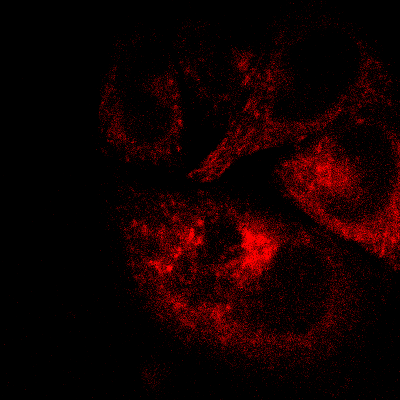

Supplement: Supplementary file 16 — Source Data [file 41467_2022_32071_MOESM16_ESM.zip › Figure 7/whole frame T1 red.png]

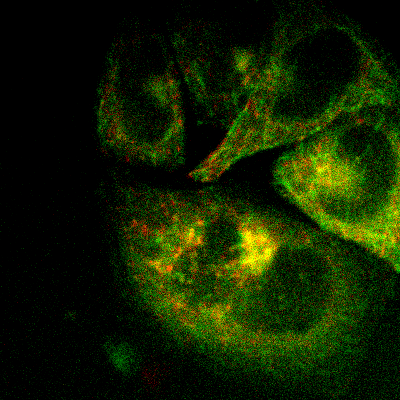

Supplement: Supplementary file 16 — Source Data [file 41467_2022_32071_MOESM16_ESM.zip › Figure 7/whole frame T1 vs T3.png]

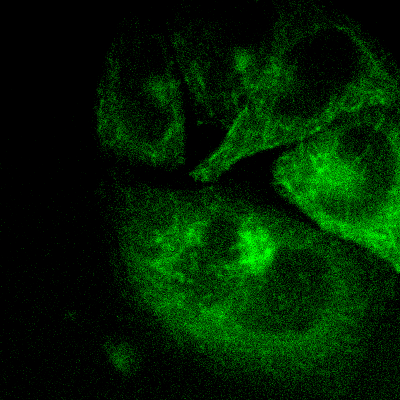

Supplement: Supplementary file 16 — Source Data [file 41467_2022_32071_MOESM16_ESM.zip › Figure 7/whole frame T3 green.png]

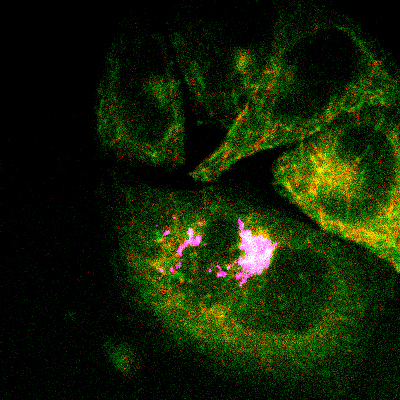

Supplement: Supplementary file 16 — Source Data [file 41467_2022_32071_MOESM16_ESM.zip › Figure 7/whole frame T3 vs T4 and APX.png]

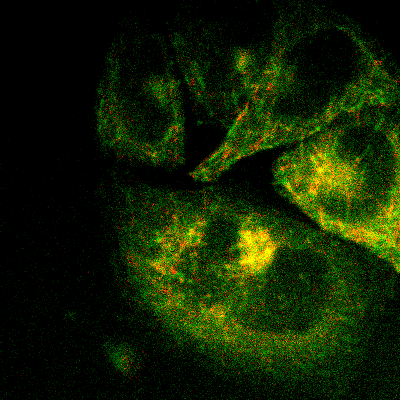

Supplement: Supplementary file 16 — Source Data [file 41467_2022_32071_MOESM16_ESM.zip › Figure 7/whole frame T3 vs T4.png]

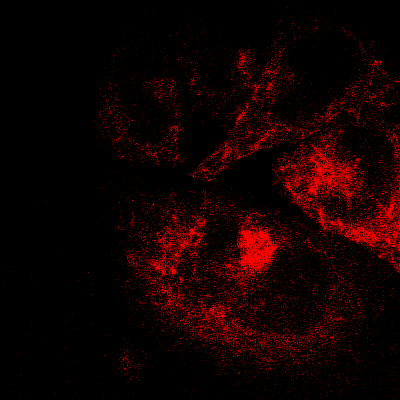

Supplement: Supplementary file 16 — Source Data [file 41467_2022_32071_MOESM16_ESM.zip › Figure 7/whole frame T4 red.png]

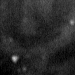

Supplement: Supplementary file 16 — Source Data [file 41467_2022_32071_MOESM16_ESM.zip › Figure 8/#13 area 2 SRS frame 1 cropped.png]

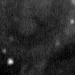

Supplement: Supplementary file 16 — Source Data [file 41467_2022_32071_MOESM16_ESM.zip › Figure 8/#13 area 2 SRS frame 100 cropped.png]

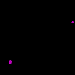

Supplement: Supplementary file 16 — Source Data [file 41467_2022_32071_MOESM16_ESM.zip › Figure 8/#13 area 2 SRS frame 101 cropped APX.png]

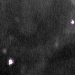

Supplement: Supplementary file 16 — Source Data [file 41467_2022_32071_MOESM16_ESM.zip › Figure 8/#13 area 2 SRS frame 101 cropped.png]

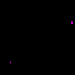

Supplement: Supplementary file 16 — Source Data [file 41467_2022_32071_MOESM16_ESM.zip › Figure 8/#13 area 2 SRS frame 120 cropped APX.png]

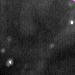

Supplement: Supplementary file 16 — Source Data [file 41467_2022_32071_MOESM16_ESM.zip › Figure 8/#13 area 2 SRS frame 120 cropped.png]

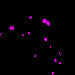

Supplement: Supplementary file 16 — Source Data [file 41467_2022_32071_MOESM16_ESM.zip › Figure 8/#13 area 2 SRS frame 140 cropped APX.png]

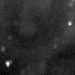

Supplement: Supplementary file 16 — Source Data [file 41467_2022_32071_MOESM16_ESM.zip › Figure 8/#13 area 2 SRS frame 140 cropped.png]

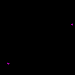

Supplement: Supplementary file 16 — Source Data [file 41467_2022_32071_MOESM16_ESM.zip › Figure 8/#13 area 2 SRS frame 160 cropped APX.png]

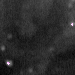

Supplement: Supplementary file 16 — Source Data [file 41467_2022_32071_MOESM16_ESM.zip › Figure 8/#13 area 2 SRS frame 160 cropped.png]

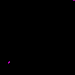

Supplement: Supplementary file 16 — Source Data [file 41467_2022_32071_MOESM16_ESM.zip › Figure 8/#13 area 2 SRS frame 180 cropped APX.png]

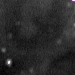

Supplement: Supplementary file 16 — Source Data [file 41467_2022_32071_MOESM16_ESM.zip › Figure 8/#13 area 2 SRS frame 180 cropped.png]

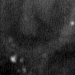

Supplement: Supplementary file 16 — Source Data [file 41467_2022_32071_MOESM16_ESM.zip › Figure 8/#13 area 2 SRS frame 20 cropped.png]

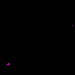

Supplement: Supplementary file 16 — Source Data [file 41467_2022_32071_MOESM16_ESM.zip › Figure 8/#13 area 2 SRS frame 200 cropped APX.png]

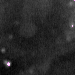

Supplement: Supplementary file 16 — Source Data [file 41467_2022_32071_MOESM16_ESM.zip › Figure 8/#13 area 2 SRS frame 200 cropped.png]

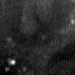

Supplement: Supplementary file 16 — Source Data [file 41467_2022_32071_MOESM16_ESM.zip › Figure 8/#13 area 2 SRS frame 40 cropped.png]

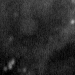

Supplement: Supplementary file 16 — Source Data [file 41467_2022_32071_MOESM16_ESM.zip › Figure 8/#13 area 2 SRS frame 60 cropped.png]

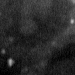

Supplement: Supplementary file 16 — Source Data [file 41467_2022_32071_MOESM16_ESM.zip › Figure 8/#13 area 2 SRS frame 80 cropped.png]

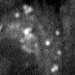

Supplement: Supplementary file 16 — Source Data [file 41467_2022_32071_MOESM16_ESM.zip › Figure 8/#13 SRS frame 1 cropped.png]

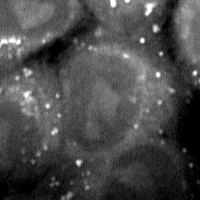

Supplement: Supplementary file 16 — Source Data [file 41467_2022_32071_MOESM16_ESM.zip › Figure 8/#13 SRS frame 1.1.png]

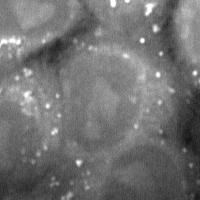

Supplement: Supplementary file 16 — Source Data [file 41467_2022_32071_MOESM16_ESM.zip › Figure 8/#13 SRS frame 1.png]

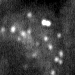

Supplement: Supplementary file 16 — Source Data [file 41467_2022_32071_MOESM16_ESM.zip › Figure 8/#13 SRS frame 100 cropped.png]

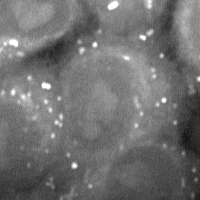

Supplement: Supplementary file 16 — Source Data [file 41467_2022_32071_MOESM16_ESM.zip › Figure 8/#13 SRS frame 100.png]

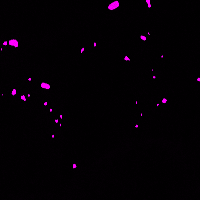

Supplement: Supplementary file 16 — Source Data [file 41467_2022_32071_MOESM16_ESM.zip › Figure 8/#13 SRS frame 101 APX.png]

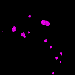

Supplement: Supplementary file 16 — Source Data [file 41467_2022_32071_MOESM16_ESM.zip › Figure 8/#13 SRS frame 101 cropped APX.png]

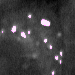

Supplement: Supplementary file 16 — Source Data [file 41467_2022_32071_MOESM16_ESM.zip › Figure 8/#13 SRS frame 101 cropped.png]

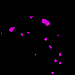

Supplement: Supplementary file 16 — Source Data [file 41467_2022_32071_MOESM16_ESM.zip › Figure 8/#13 SRS frame 120 cropped APX.png]

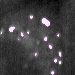

Supplement: Supplementary file 16 — Source Data [file 41467_2022_32071_MOESM16_ESM.zip › Figure 8/#13 SRS frame 120 cropped.png]

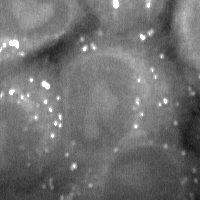

Supplement: Supplementary file 16 — Source Data [file 41467_2022_32071_MOESM16_ESM.zip › Figure 8/#13 SRS frame 120.png]

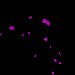

Supplement: Supplementary file 16 — Source Data [file 41467_2022_32071_MOESM16_ESM.zip › Figure 8/#13 SRS frame 140 cropped APX.png]

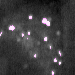

Supplement: Supplementary file 16 — Source Data [file 41467_2022_32071_MOESM16_ESM.zip › Figure 8/#13 SRS frame 140 cropped.png]

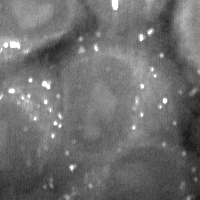

Supplement: Supplementary file 16 — Source Data [file 41467_2022_32071_MOESM16_ESM.zip › Figure 8/#13 SRS frame 140.png]

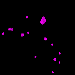

Supplement: Supplementary file 16 — Source Data [file 41467_2022_32071_MOESM16_ESM.zip › Figure 8/#13 SRS frame 160 cropped APX.png]

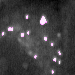

Supplement: Supplementary file 16 — Source Data [file 41467_2022_32071_MOESM16_ESM.zip › Figure 8/#13 SRS frame 160 cropped.png]

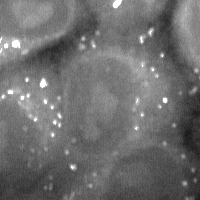

Supplement: Supplementary file 16 — Source Data [file 41467_2022_32071_MOESM16_ESM.zip › Figure 8/#13 SRS frame 160.png]

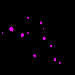

Supplement: Supplementary file 16 — Source Data [file 41467_2022_32071_MOESM16_ESM.zip › Figure 8/#13 SRS frame 180 cropped APX.png]

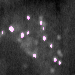

Supplement: Supplementary file 16 — Source Data [file 41467_2022_32071_MOESM16_ESM.zip › Figure 8/#13 SRS frame 180 cropped.png]

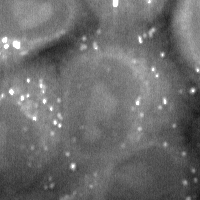

Supplement: Supplementary file 16 — Source Data [file 41467_2022_32071_MOESM16_ESM.zip › Figure 8/#13 SRS frame 180.png]

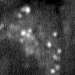

Supplement: Supplementary file 16 — Source Data [file 41467_2022_32071_MOESM16_ESM.zip › Figure 8/#13 SRS frame 20 cropped.png]

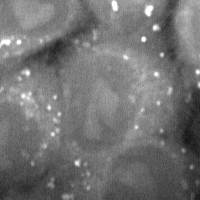

Supplement: Supplementary file 16 — Source Data [file 41467_2022_32071_MOESM16_ESM.zip › Figure 8/#13 SRS frame 20.png]

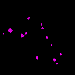

Supplement: Supplementary file 16 — Source Data [file 41467_2022_32071_MOESM16_ESM.zip › Figure 8/#13 SRS frame 200 cropped APX.png]

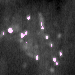

Supplement: Supplementary file 16 — Source Data [file 41467_2022_32071_MOESM16_ESM.zip › Figure 8/#13 SRS frame 200 cropped.png]

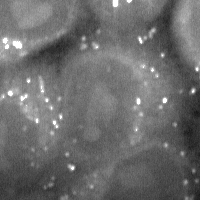

Supplement: Supplementary file 16 — Source Data [file 41467_2022_32071_MOESM16_ESM.zip › Figure 8/#13 SRS frame 200.png]

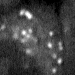

Supplement: Supplementary file 16 — Source Data [file 41467_2022_32071_MOESM16_ESM.zip › Figure 8/#13 SRS frame 40 cropped.png]

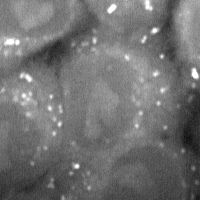

Supplement: Supplementary file 16 — Source Data [file 41467_2022_32071_MOESM16_ESM.zip › Figure 8/#13 SRS frame 40.png]

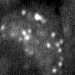

Supplement: Supplementary file 16 — Source Data [file 41467_2022_32071_MOESM16_ESM.zip › Figure 8/#13 SRS frame 60 cropped.png]

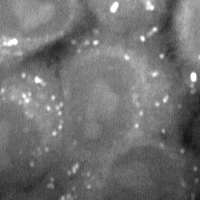

Supplement: Supplementary file 16 — Source Data [file 41467_2022_32071_MOESM16_ESM.zip › Figure 8/#13 SRS frame 60.png]

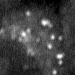

Supplement: Supplementary file 16 — Source Data [file 41467_2022_32071_MOESM16_ESM.zip › Figure 8/#13 SRS frame 80 cropped.png]

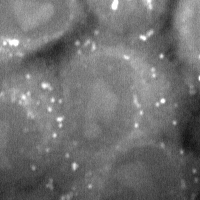

Supplement: Supplementary file 16 — Source Data [file 41467_2022_32071_MOESM16_ESM.zip › Figure 8/#13 SRS frame 80.png]
