# Supplementary figures and images for: Real-time precision opto-control of chemical processes in live cells (part 1 of 2)
Source: Nat Commun. 2022 Jul 27;13:4343. doi: 10.1038/s41467-022-32071-z (PMC9329476; doi:10.1038/s41467-022-32071-z)

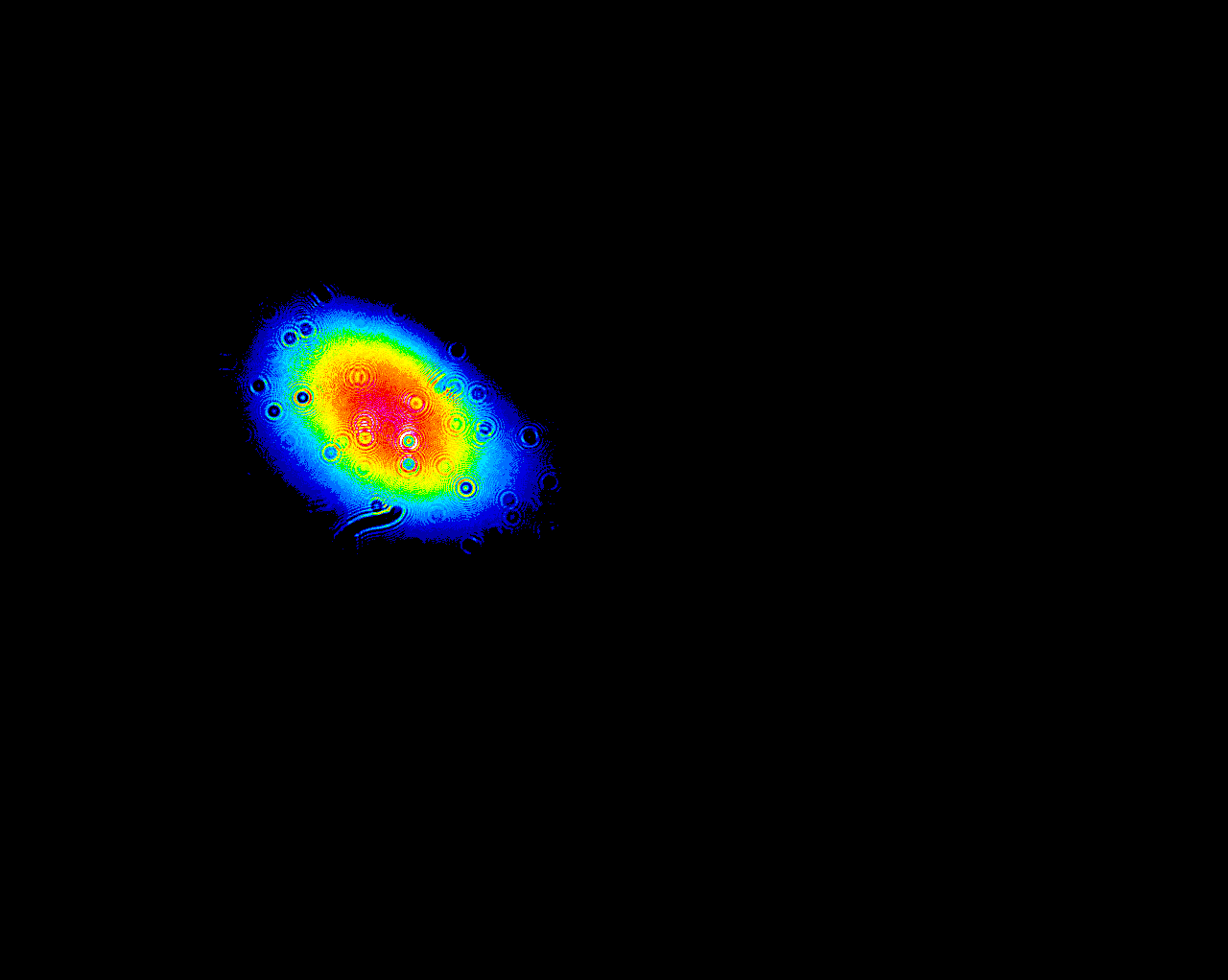

Supplement: Supplementary file 16 — Source Data [file 41467_2022_32071_MOESM16_ESM.zip › Figure 1/beamprofile0order.png]

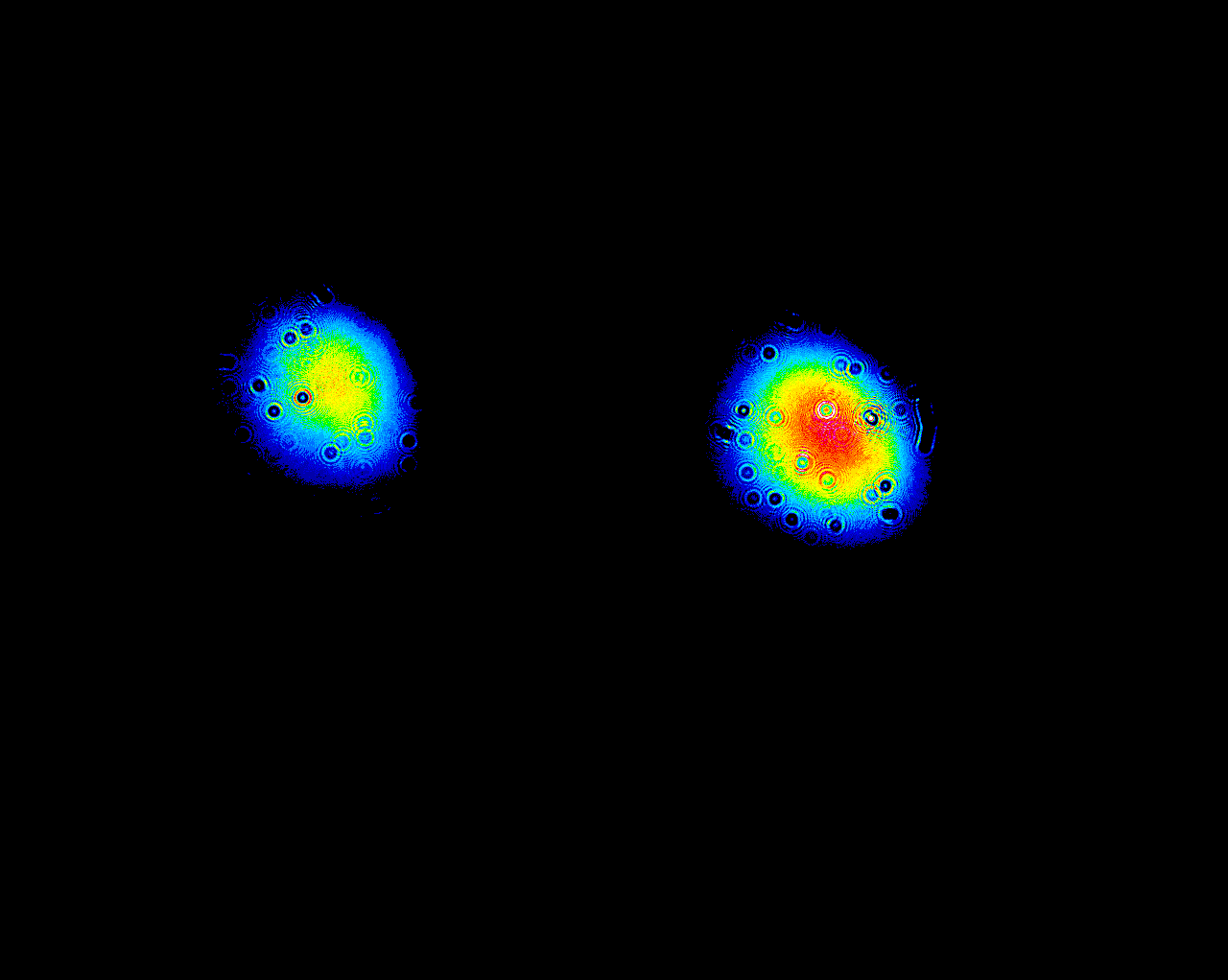

Supplement: Supplementary file 16 — Source Data [file 41467_2022_32071_MOESM16_ESM.zip › Figure 1/beamprofilebothbeams.png]

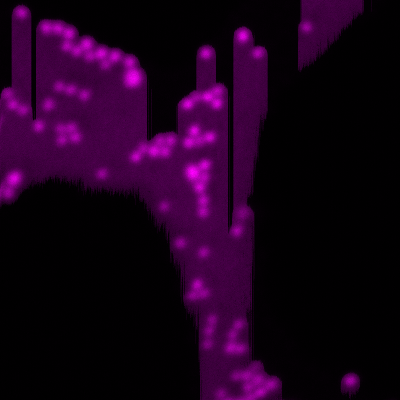

Supplement: Supplementary file 16 — Source Data [file 41467_2022_32071_MOESM16_ESM.zip › Figure 2/## 1045 nm 570 nm channel 0.5 V low magenta.png]

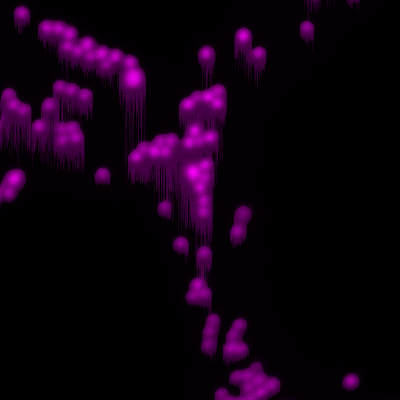

Supplement: Supplementary file 16 — Source Data [file 41467_2022_32071_MOESM16_ESM.zip › Figure 2/## 1045 nm 570 nm channel 0.72 V low magenta.png]

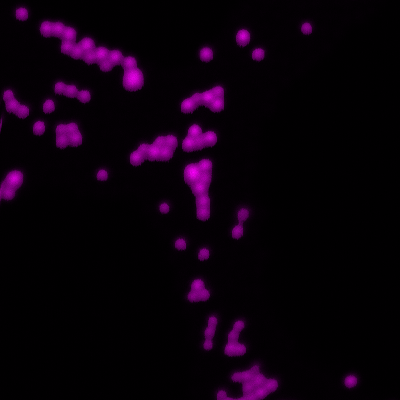

Supplement: Supplementary file 16 — Source Data [file 41467_2022_32071_MOESM16_ESM.zip › Figure 2/## 1045 nm 570 nm channel 1.2 V optimal magenta.png]

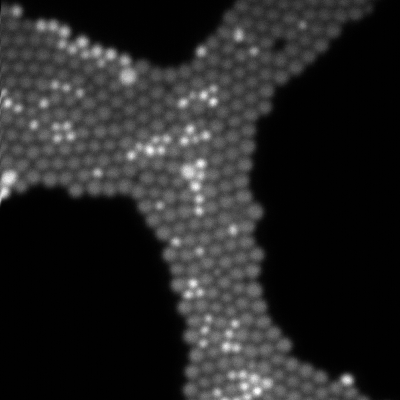

Supplement: Supplementary file 16 — Source Data [file 41467_2022_32071_MOESM16_ESM.zip › Figure 2/## 800 nm ex 570 nm channel.png]

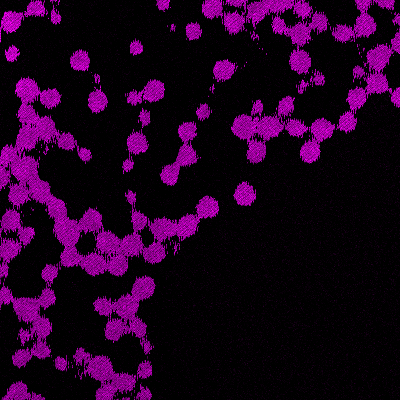

Supplement: Supplementary file 16 — Source Data [file 41467_2022_32071_MOESM16_ESM.zip › Figure 2/0.4 V first peak magenta.png]

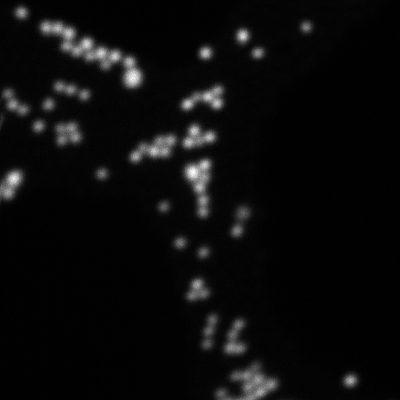

Supplement: Supplementary file 16 — Source Data [file 41467_2022_32071_MOESM16_ESM.zip › Figure 2/1045 nm ex 570 nm channel.png]

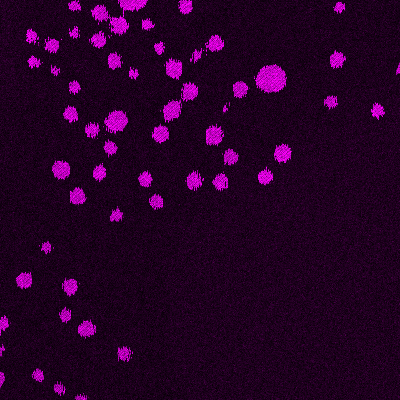

Supplement: Supplementary file 16 — Source Data [file 41467_2022_32071_MOESM16_ESM.zip › Figure 2/beads SRS 2955 peak.png]

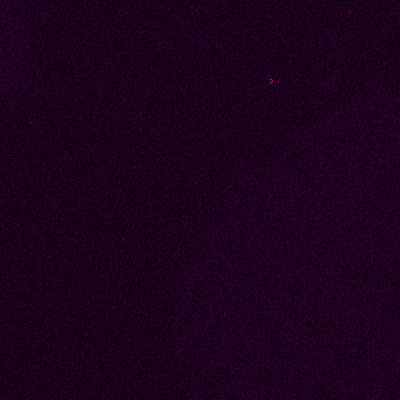

Supplement: Supplementary file 16 — Source Data [file 41467_2022_32071_MOESM16_ESM.zip › Figure 2/beads SRS 2990 no peak.png]

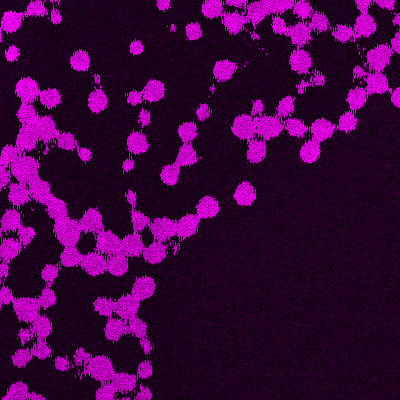

Supplement: Supplementary file 16 — Source Data [file 41467_2022_32071_MOESM16_ESM.zip › Figure 2/beads SRS 3060 peak.png]

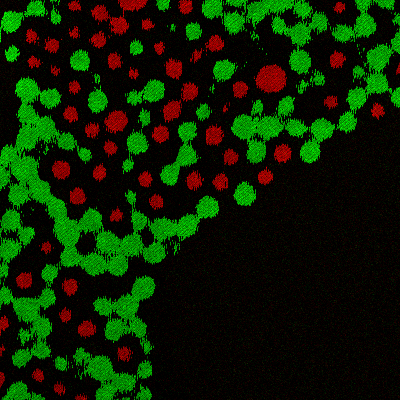

Supplement: Supplementary file 16 — Source Data [file 41467_2022_32071_MOESM16_ESM.zip › Figure 2/Composite 0.4 V image both peaks.png]

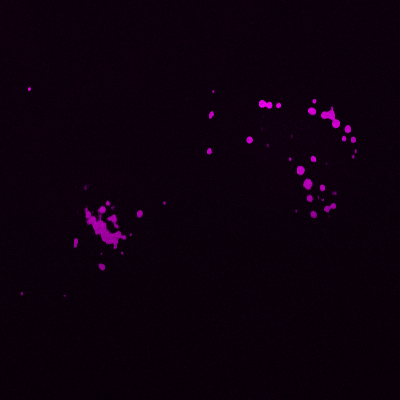

Supplement: Supplementary file 16 — Source Data [file 41467_2022_32071_MOESM16_ESM.zip › Figure 2/Mia PaCa2 0.7 V transmission magenta.png]

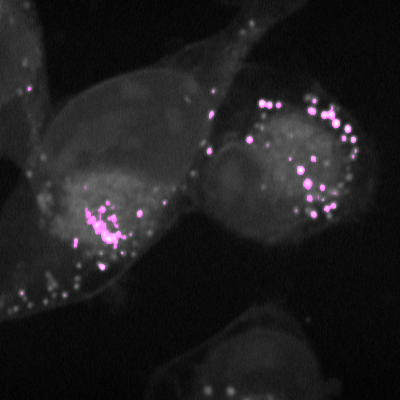

Supplement: Supplementary file 16 — Source Data [file 41467_2022_32071_MOESM16_ESM.zip › Figure 2/Mia PaCa2 30 mW 100 mW 0.6 SRS overlap.png]

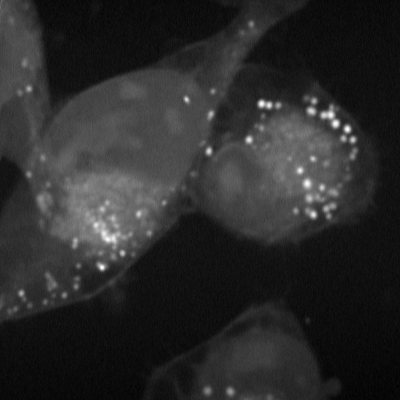

Supplement: Supplementary file 16 — Source Data [file 41467_2022_32071_MOESM16_ESM.zip › Figure 2/Mia PaCa2 30 mW 100 mW 0.6 SRS.png]

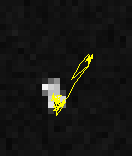

Supplement: Supplementary file 16 — Source Data [file 41467_2022_32071_MOESM16_ESM.zip › Figure 2/Trajectory number APX.png]

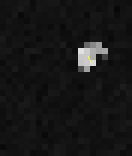

Supplement: Supplementary file 16 — Source Data [file 41467_2022_32071_MOESM16_ESM.zip › Figure 2/Trajectory number APX-1.png]

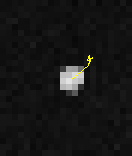

Supplement: Supplementary file 16 — Source Data [file 41467_2022_32071_MOESM16_ESM.zip › Figure 2/Trajectory number APX-11.png]

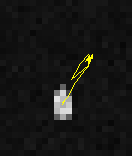

Supplement: Supplementary file 16 — Source Data [file 41467_2022_32071_MOESM16_ESM.zip › Figure 2/Trajectory number APX-31.png]

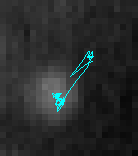

Supplement: Supplementary file 16 — Source Data [file 41467_2022_32071_MOESM16_ESM.zip › Figure 2/Trajectory number LD.png]

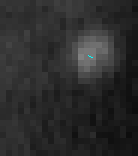

Supplement: Supplementary file 16 — Source Data [file 41467_2022_32071_MOESM16_ESM.zip › Figure 2/Trajectory number LD-1.png]

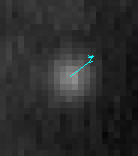

Supplement: Supplementary file 16 — Source Data [file 41467_2022_32071_MOESM16_ESM.zip › Figure 2/Trajectory number LD-11.png]

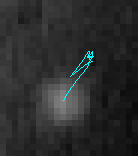

Supplement: Supplementary file 16 — Source Data [file 41467_2022_32071_MOESM16_ESM.zip › Figure 2/Trajectory number LD-31.png]

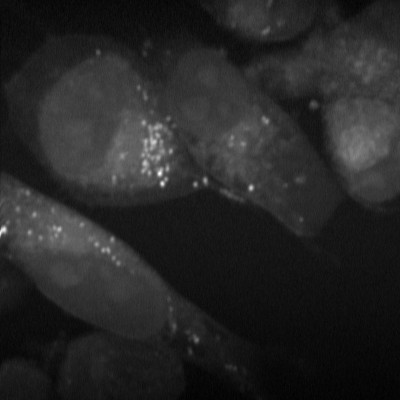

Supplement: Supplementary file 16 — Source Data [file 41467_2022_32071_MOESM16_ESM.zip › Figure 3/AVG_Stack.png]

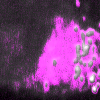

Supplement: Supplementary file 16 — Source Data [file 41467_2022_32071_MOESM16_ESM.zip › Figure 3/Composite 1.png]

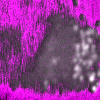

Supplement: Supplementary file 16 — Source Data [file 41467_2022_32071_MOESM16_ESM.zip › Figure 3/Composite 2.png]

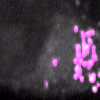

Supplement: Supplementary file 16 — Source Data [file 41467_2022_32071_MOESM16_ESM.zip › Figure 3/Composite 3.png]

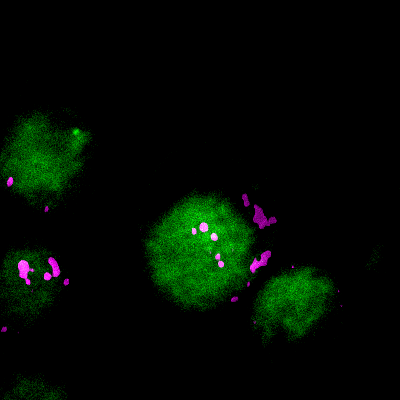

Supplement: Supplementary file 16 — Source Data [file 41467_2022_32071_MOESM16_ESM.zip › Figure 3/ER and 0.25 0.1V.png]

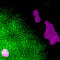

Supplement: Supplementary file 16 — Source Data [file 41467_2022_32071_MOESM16_ESM.zip › Figure 3/ER and 0.25 0.1V-1.png]

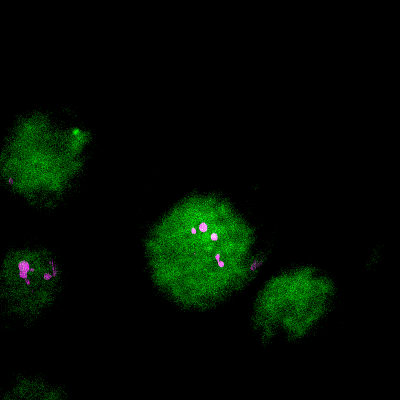

Supplement: Supplementary file 16 — Source Data [file 41467_2022_32071_MOESM16_ESM.zip › Figure 3/ER and 0.25 0.25V.png]

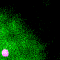

Supplement: Supplementary file 16 — Source Data [file 41467_2022_32071_MOESM16_ESM.zip › Figure 3/ER and 0.25 0.25V-1.png]

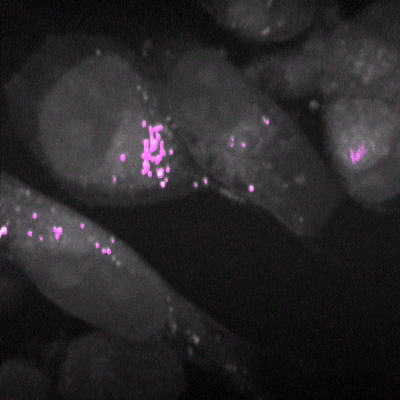

Supplement: Supplementary file 16 — Source Data [file 41467_2022_32071_MOESM16_ESM.zip › Figure 3/MIA PaCa 30 mW 100 mW one box B1 0.28 0.004 60x no LD 10 frames composit.png]

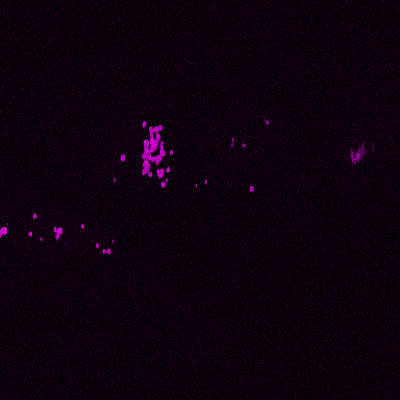

Supplement: Supplementary file 16 — Source Data [file 41467_2022_32071_MOESM16_ESM.zip › Figure 3/MIA PaCa 30 mW 100 mW one box B1 0.28 0.004 60x no LD 10 frames.png]

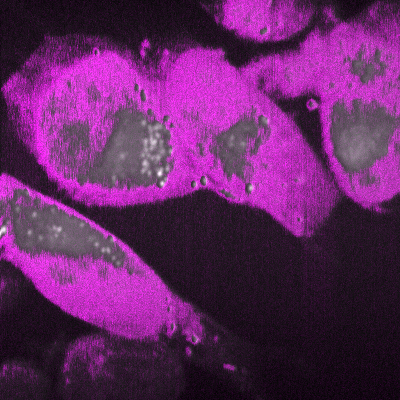

Supplement: Supplementary file 16 — Source Data [file 41467_2022_32071_MOESM16_ESM.zip › Figure 3/MIA PaCa 30mW 100mW both box B1 0.16 B2 0.1 0.004 60x no LD 10 frames composit.png]

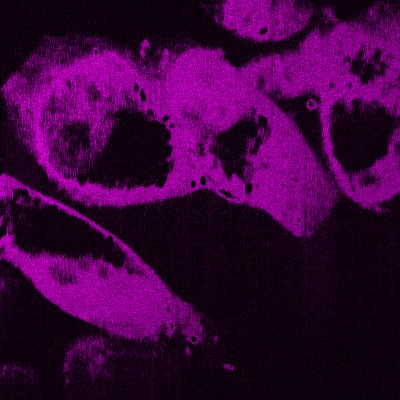

Supplement: Supplementary file 16 — Source Data [file 41467_2022_32071_MOESM16_ESM.zip › Figure 3/MIA PaCa 30mW 100mW both box B1 0.16 B2 0.1 0.004 60x no LD 10 frames.png]

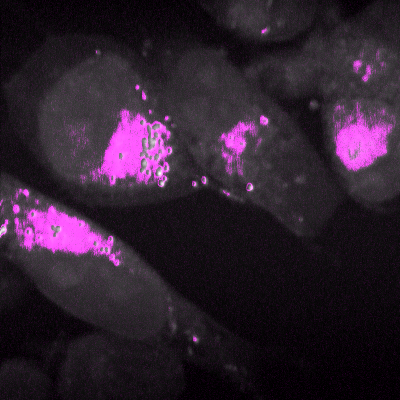

Supplement: Supplementary file 16 — Source Data [file 41467_2022_32071_MOESM16_ESM.zip › Figure 3/MIA PaCa 30mW 100mW both box B1 0.3 B2 0.2 0.004 60x no LD 10 frames composit.png]

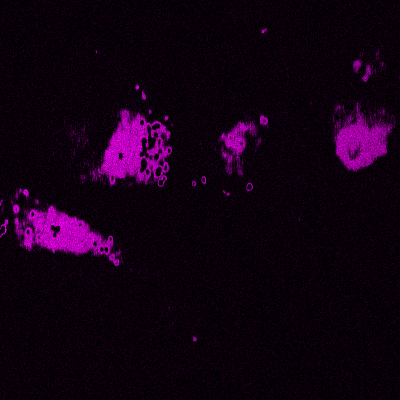

Supplement: Supplementary file 16 — Source Data [file 41467_2022_32071_MOESM16_ESM.zip › Figure 3/MIA PaCa 30mW 100mW both box B1 0.3 B2 0.2 0.004 60x no LD 10 frames.png]

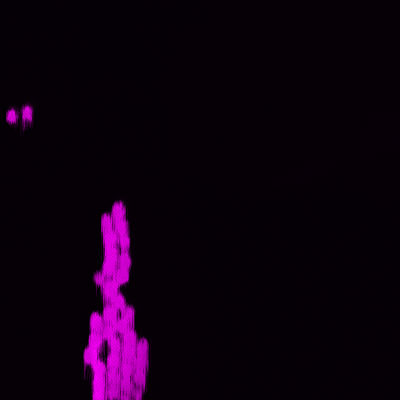

Supplement: Supplementary file 16 — Source Data [file 41467_2022_32071_MOESM16_ESM.zip › Figure 3/PS PSF NADH 30mW 60mW 60x 0.003 PMT 450nm 0.02V 0.125V.png]

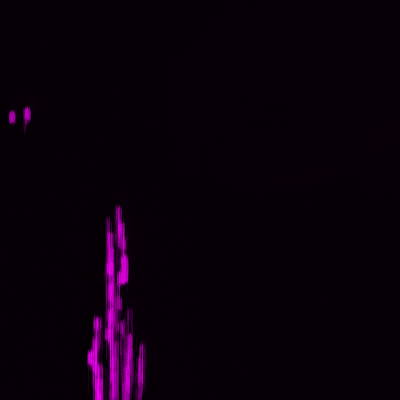

Supplement: Supplementary file 16 — Source Data [file 41467_2022_32071_MOESM16_ESM.zip › Figure 3/PS PSF NADH 30mW 60mW 60x 0.003 PMT 450nm 0.02V 0.25V.png]

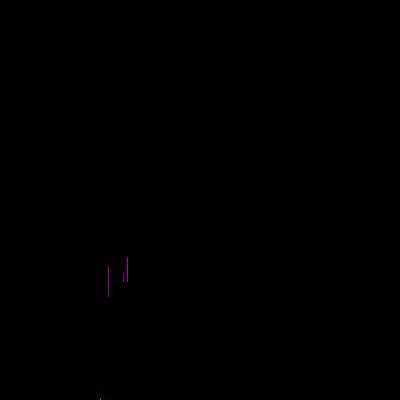

Supplement: Supplementary file 16 — Source Data [file 41467_2022_32071_MOESM16_ESM.zip › Figure 3/PS PSF NADH 30mW 60mW 60x 0.003 PMT 450nm 0.02V 0.375V.png]

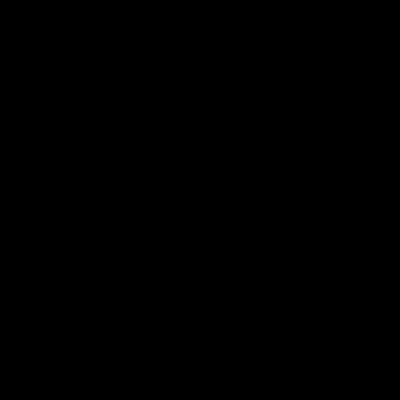

Supplement: Supplementary file 16 — Source Data [file 41467_2022_32071_MOESM16_ESM.zip › Figure 3/PS PSF NADH 30mW 60mW 60x 0.003 PMT 450nm 0.02V 0.5V.png]

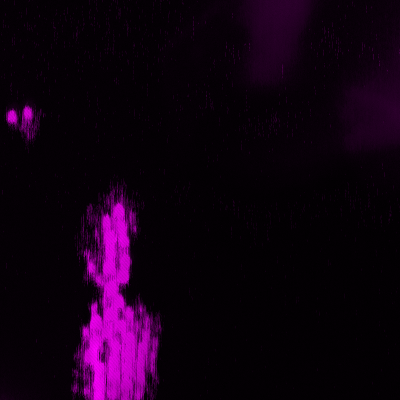

Supplement: Supplementary file 16 — Source Data [file 41467_2022_32071_MOESM16_ESM.zip › Figure 3/PS PSF NADH 30mW 60mW 60x 0.003 PMT 450nm 0.02V 0V.png]

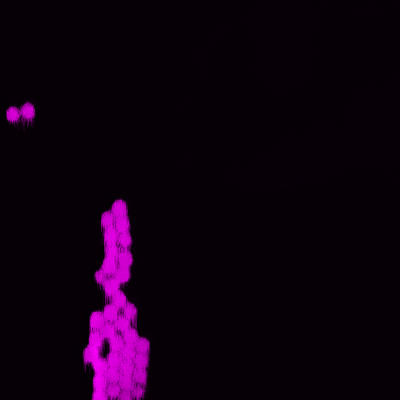

Supplement: Supplementary file 16 — Source Data [file 41467_2022_32071_MOESM16_ESM.zip › Figure 3/PS PSF NADH 30mW 60mW 60x 0.003 PMT 450nm 0.05V 0.125V.png]

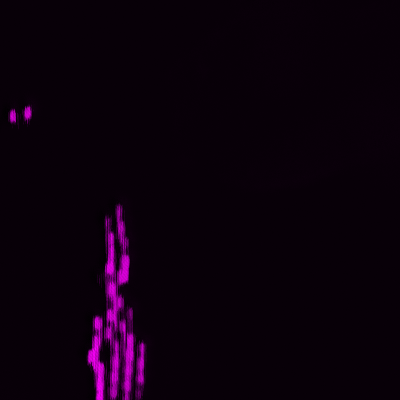

Supplement: Supplementary file 16 — Source Data [file 41467_2022_32071_MOESM16_ESM.zip › Figure 3/PS PSF NADH 30mW 60mW 60x 0.003 PMT 450nm 0.05V 0.25V.png]

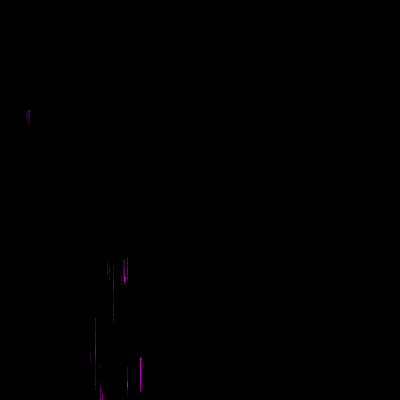

Supplement: Supplementary file 16 — Source Data [file 41467_2022_32071_MOESM16_ESM.zip › Figure 3/PS PSF NADH 30mW 60mW 60x 0.003 PMT 450nm 0.05V 0.375V.png]

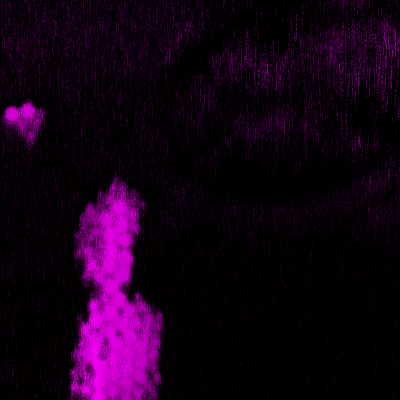

Supplement: Supplementary file 16 — Source Data [file 41467_2022_32071_MOESM16_ESM.zip › Figure 3/PS PSF NADH 30mW 60mW 60x 0.003 PMT 450nm 0.05V 0V.png]

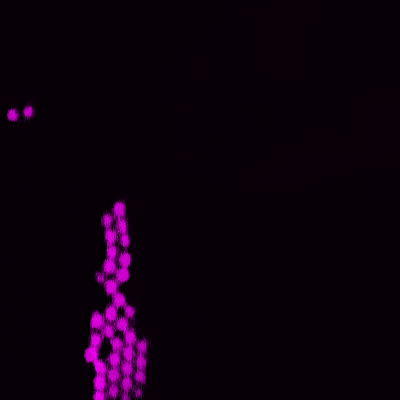

Supplement: Supplementary file 16 — Source Data [file 41467_2022_32071_MOESM16_ESM.zip › Figure 3/PS PSF NADH 30mW 60mW 60x 0.003 PMT 450nm 0.08V 0.125V.png]

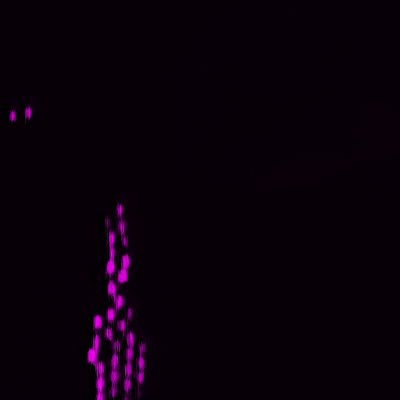

Supplement: Supplementary file 16 — Source Data [file 41467_2022_32071_MOESM16_ESM.zip › Figure 3/PS PSF NADH 30mW 60mW 60x 0.003 PMT 450nm 0.08V 0.25V.png]

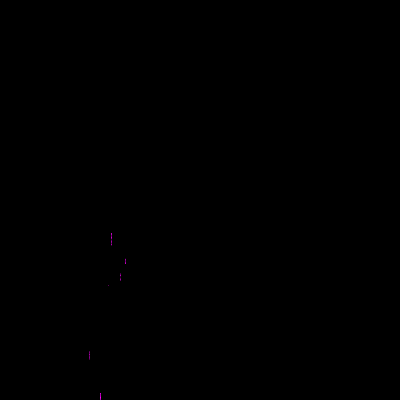

Supplement: Supplementary file 16 — Source Data [file 41467_2022_32071_MOESM16_ESM.zip › Figure 3/PS PSF NADH 30mW 60mW 60x 0.003 PMT 450nm 0.08V 0.375V.png]

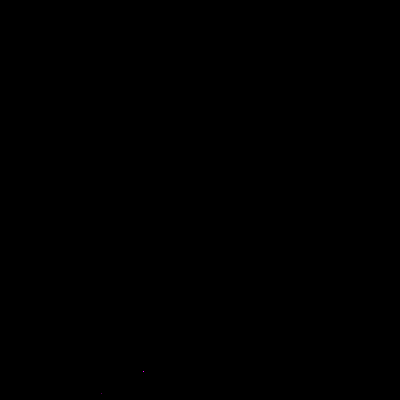

Supplement: Supplementary file 16 — Source Data [file 41467_2022_32071_MOESM16_ESM.zip › Figure 3/PS PSF NADH 30mW 60mW 60x 0.003 PMT 450nm 0.08V 0.5V.png]

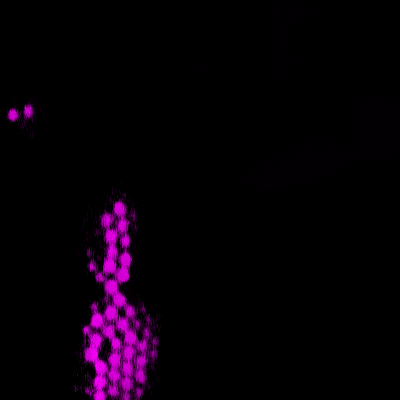

Supplement: Supplementary file 16 — Source Data [file 41467_2022_32071_MOESM16_ESM.zip › Figure 3/PS PSF NADH 30mW 60mW 60x 0.003 PMT 450nm 0.08V 0V.png]

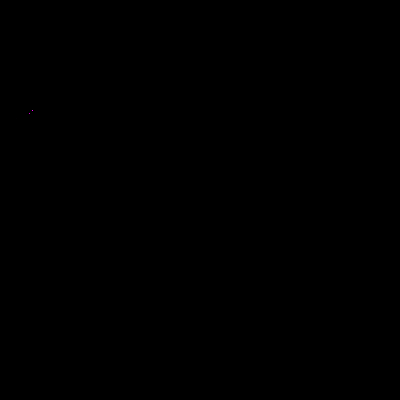

Supplement: Supplementary file 16 — Source Data [file 41467_2022_32071_MOESM16_ESM.zip › Figure 3/PS PSF NADH 30mW 60mW 60x 0.003 PMT 450nm 0.1V 0.375V.png]

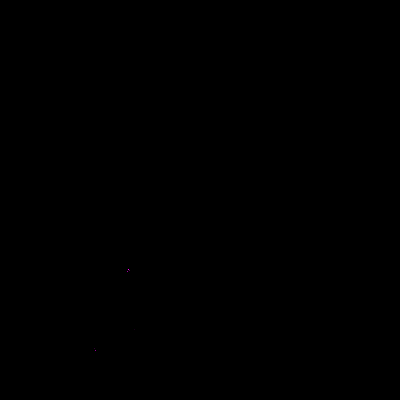

Supplement: Supplementary file 16 — Source Data [file 41467_2022_32071_MOESM16_ESM.zip › Figure 3/PS PSF NADH 30mW 60mW 60x 0.003 PMT 450nm 0.1V 0.5V.png]

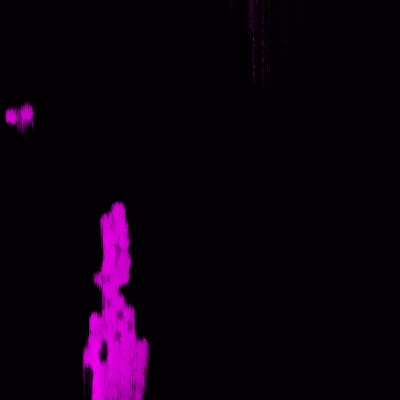

Supplement: Supplementary file 16 — Source Data [file 41467_2022_32071_MOESM16_ESM.zip › Figure 3/PS PSF NADH 30mW 60mW 60x 0.003 PMT 450nm 0V 0.125V.png]

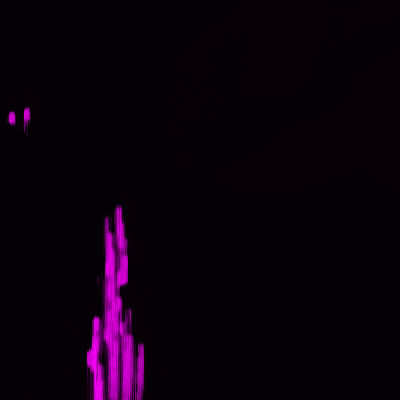

Supplement: Supplementary file 16 — Source Data [file 41467_2022_32071_MOESM16_ESM.zip › Figure 3/PS PSF NADH 30mW 60mW 60x 0.003 PMT 450nm 0V 0.25V.png]

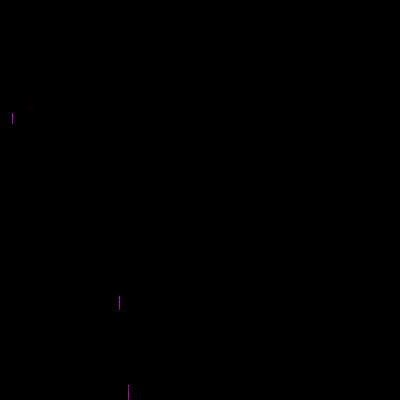

Supplement: Supplementary file 16 — Source Data [file 41467_2022_32071_MOESM16_ESM.zip › Figure 3/PS PSF NADH 30mW 60mW 60x 0.003 PMT 450nm 0V 0.375V.png]

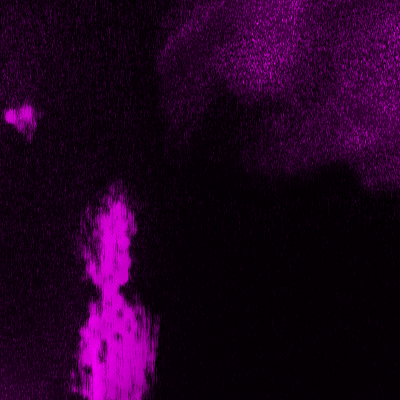

Supplement: Supplementary file 16 — Source Data [file 41467_2022_32071_MOESM16_ESM.zip › Figure 3/PS PSF NADH 30mW 60mW 60x 0.003 PMT 450nm 0V 0V.png]

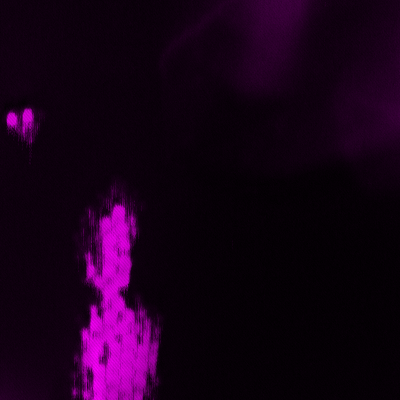

Supplement: Supplementary file 16 — Source Data [file 41467_2022_32071_MOESM16_ESM.zip › Figure 3/PS PSF NADH 30mW 60mW 60x 0.003 PMT 450nm Box1 only 0.02V.png]

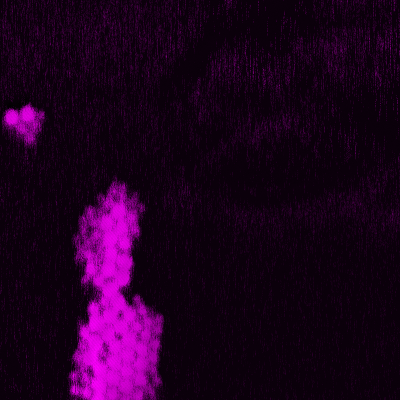

Supplement: Supplementary file 16 — Source Data [file 41467_2022_32071_MOESM16_ESM.zip › Figure 3/PS PSF NADH 30mW 60mW 60x 0.003 PMT 450nm Box1 only 0.05V.png]

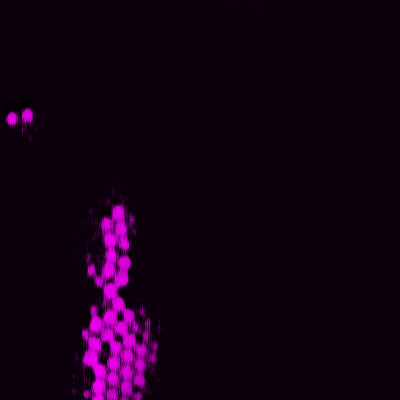

Supplement: Supplementary file 16 — Source Data [file 41467_2022_32071_MOESM16_ESM.zip › Figure 3/PS PSF NADH 30mW 60mW 60x 0.003 PMT 450nm Box1 only 0.08V.png]

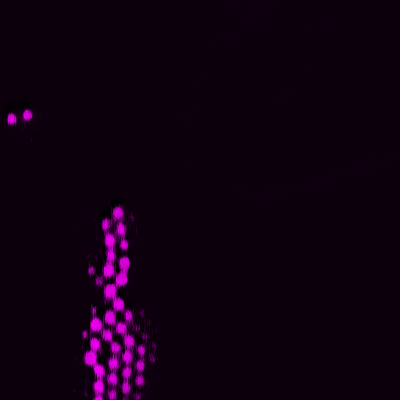

Supplement: Supplementary file 16 — Source Data [file 41467_2022_32071_MOESM16_ESM.zip › Figure 3/PS PSF NADH 30mW 60mW 60x 0.003 PMT 450nm Box1 only 0.1V.png]

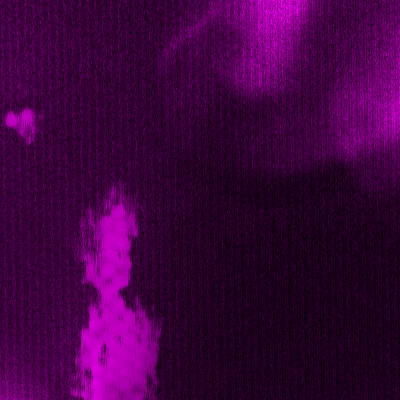

Supplement: Supplementary file 16 — Source Data [file 41467_2022_32071_MOESM16_ESM.zip › Figure 3/PS PSF NADH 30mW 60mW 60x 0.003 PMT 450nm Box1 only 0V.png]

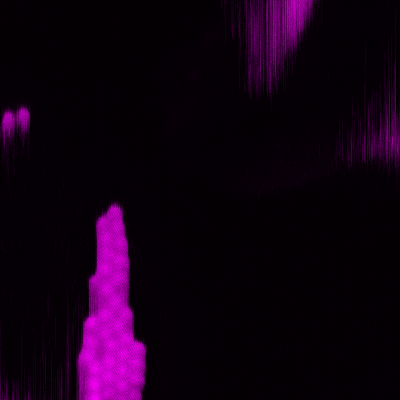

Supplement: Supplementary file 16 — Source Data [file 41467_2022_32071_MOESM16_ESM.zip › Figure 3/PS PSF NADH 30mW 60mW 60x 0.003 PMT 450nm Box1 only 450 nm 0.08V.png]

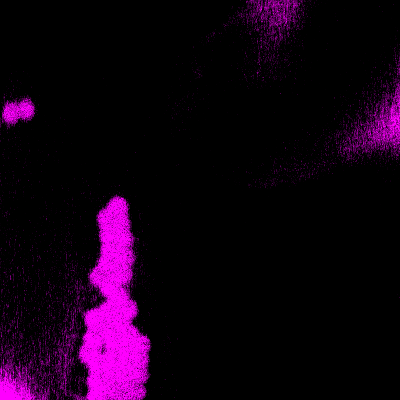

Supplement: Supplementary file 16 — Source Data [file 41467_2022_32071_MOESM16_ESM.zip › Figure 3/PS PSF NADH 30mW 60mW 60x 0.003 PMT 450nm Box1 only 450 nm 0.125V #4.png]

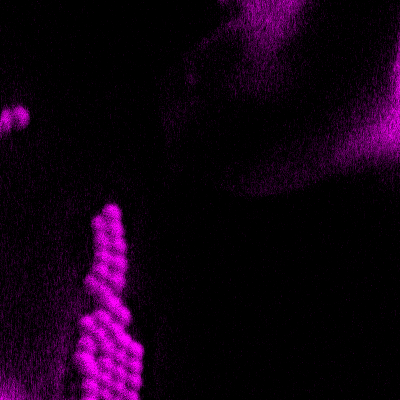

Supplement: Supplementary file 16 — Source Data [file 41467_2022_32071_MOESM16_ESM.zip › Figure 3/PS PSF NADH 30mW 60mW 60x 0.003 PMT 450nm Box1 only 450 nm 0.125V 3.png]

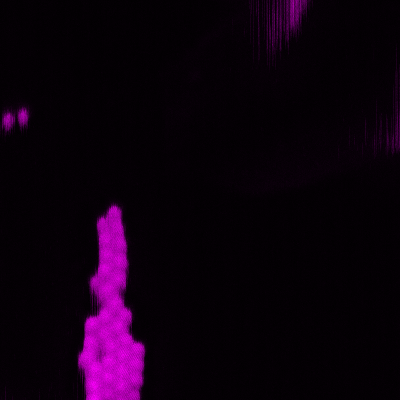

Supplement: Supplementary file 16 — Source Data [file 41467_2022_32071_MOESM16_ESM.zip › Figure 3/PS PSF NADH 30mW 60mW 60x 0.003 PMT 450nm Box1 only 450 nm 0.1V.png]

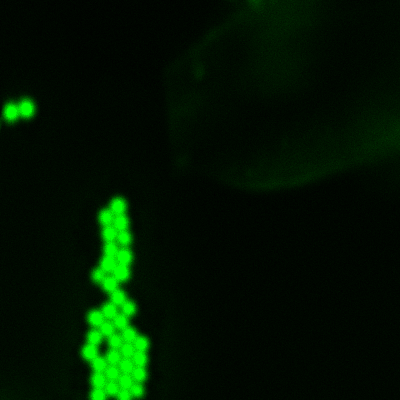

Supplement: Supplementary file 16 — Source Data [file 41467_2022_32071_MOESM16_ESM.zip › Figure 3/PS PSF NADH 30mW 60mW 60x 0.003 PMT 450nm OFF OFF.png]

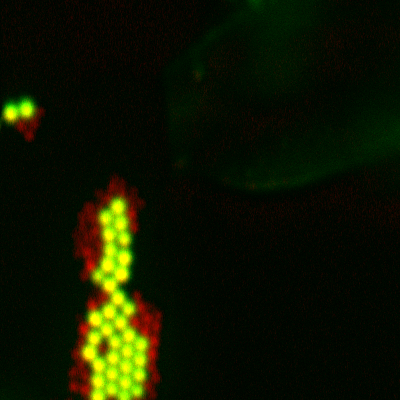

Supplement: Supplementary file 16 — Source Data [file 41467_2022_32071_MOESM16_ESM.zip › Figure 3/PS PSF NADH 30mW 60mW 60x 0.003 SRS OFF OFF and PMT 450 nm merge.png]

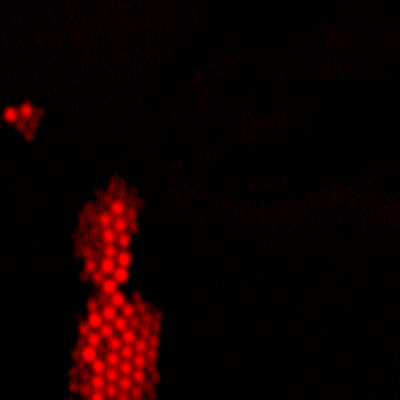

Supplement: Supplementary file 16 — Source Data [file 41467_2022_32071_MOESM16_ESM.zip › Figure 3/PS PSF NADH 30mW 60mW 60x 0.003 SRS OFF OFF.png]

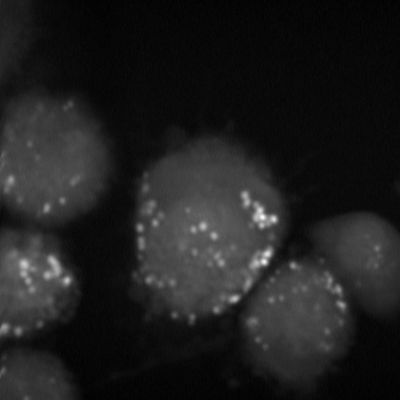

Supplement: Supplementary file 16 — Source Data [file 41467_2022_32071_MOESM16_ESM.zip › Figure 3/SRS CH #5.png]

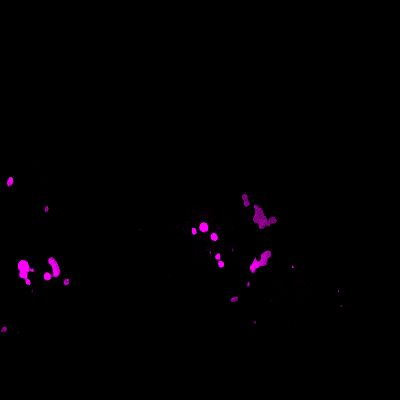

Supplement: Supplementary file 16 — Source Data [file 41467_2022_32071_MOESM16_ESM.zip › Figure 3/TPEF 30mW 40mW fs 60x 0.003 TWO BOX 0.25 0.1V #5.png]

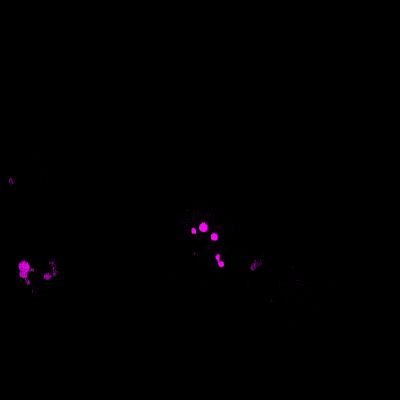

Supplement: Supplementary file 16 — Source Data [file 41467_2022_32071_MOESM16_ESM.zip › Figure 3/TPEF 30mW 40mW fs 60x 0.003 TWO BOX 0.25 0.25V #5.png]

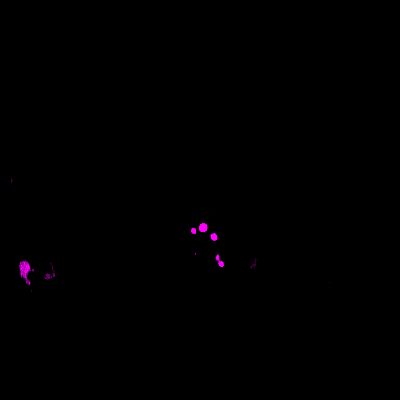

Supplement: Supplementary file 16 — Source Data [file 41467_2022_32071_MOESM16_ESM.zip › Figure 3/TPEF 30mW 40mW fs 60x 0.003 TWO BOX 0.25 0.27V #5.png]

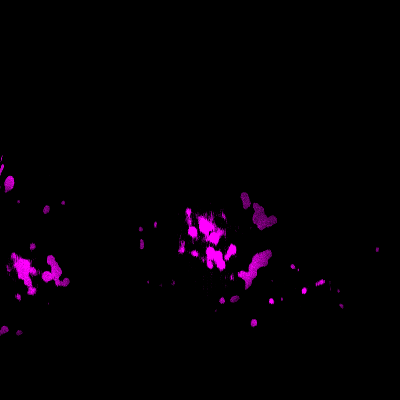

Supplement: Supplementary file 16 — Source Data [file 41467_2022_32071_MOESM16_ESM.zip › Figure 3/TPEF 30mW 40mW fs 60x 0.003 TWO BOX 0.25 0V #5.png]

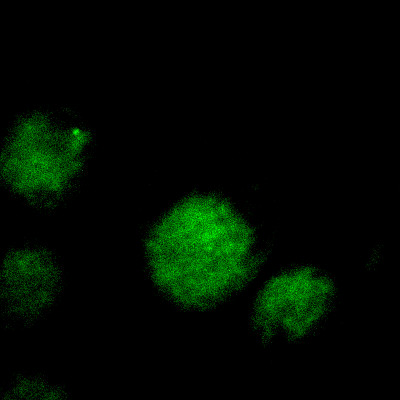

Supplement: Supplementary file 16 — Source Data [file 41467_2022_32071_MOESM16_ESM.zip › Figure 3/TPEF 30mW 40mW fs 60x 0.003 TWO BOX 0.25 0V blocked only ER #5.png]

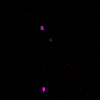

Supplement: Supplementary file 16 — Source Data [file 41467_2022_32071_MOESM16_ESM.zip › Figure 4/Mia PaCa 2 cells 30 mW 80 mW 0.003 40x obj active pixels 3 cropped.png]

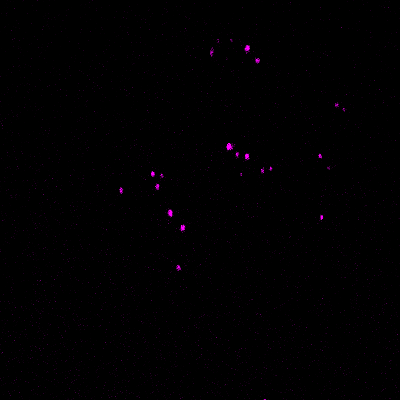

Supplement: Supplementary file 16 — Source Data [file 41467_2022_32071_MOESM16_ESM.zip › Figure 4/Mia PaCa 2 cells 30 mW 80 mW 0.003 40x obj active pixels 3.png]

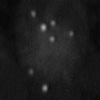

Supplement: Supplementary file 16 — Source Data [file 41467_2022_32071_MOESM16_ESM.zip › Figure 4/Mia PaCa 2 cells 30 mW 80 mW 0.003 40x obj CH cropped.png]

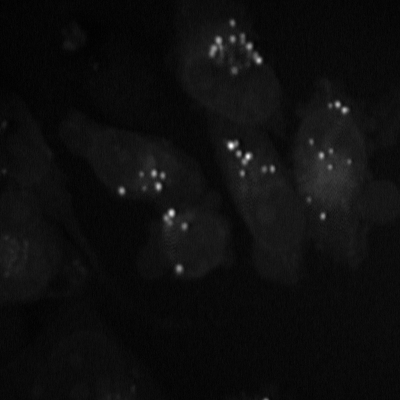

Supplement: Supplementary file 16 — Source Data [file 41467_2022_32071_MOESM16_ESM.zip › Figure 4/Mia PaCa 2 cells 30 mW 80 mW 0.003 40x obj CH.png]

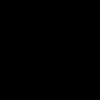

Supplement: Supplementary file 16 — Source Data [file 41467_2022_32071_MOESM16_ESM.zip › Figure 4/Mia PaCa 2 cells 30 mW 80 mW 0.003 40x obj image fully open 3 cropped.png]

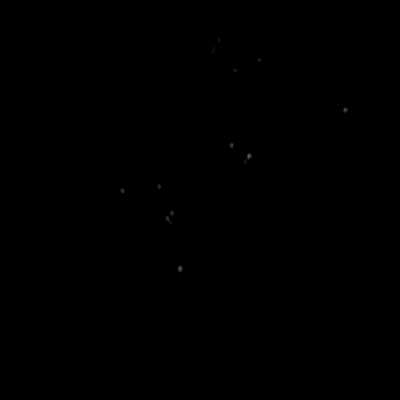

Supplement: Supplementary file 16 — Source Data [file 41467_2022_32071_MOESM16_ESM.zip › Figure 4/Mia PaCa 2 cells 30 mW 80 mW 0.003 40x obj image fully open 3.png]

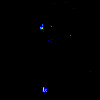

Supplement: Supplementary file 16 — Source Data [file 41467_2022_32071_MOESM16_ESM.zip › Figure 4/Mia PaCa 2 cells 30 mW 80 mW 0.003 40x obj image subtract 3 cropped.png]

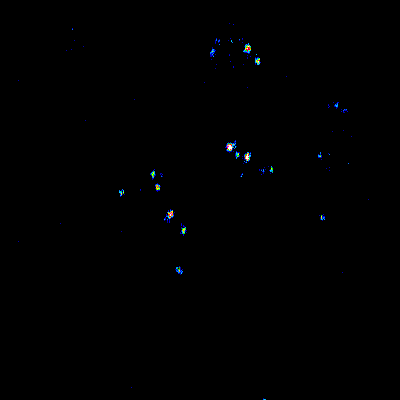

Supplement: Supplementary file 16 — Source Data [file 41467_2022_32071_MOESM16_ESM.zip › Figure 4/Mia PaCa 2 cells 30 mW 80 mW 0.003 40x obj image subtract 3.png]

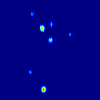

Supplement: Supplementary file 16 — Source Data [file 41467_2022_32071_MOESM16_ESM.zip › Figure 4/Mia PaCa 2 cells 30 mW 80 mW 0.003 40x obj SRS blocked 3 cropped color.png]

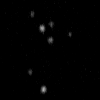

Supplement: Supplementary file 16 — Source Data [file 41467_2022_32071_MOESM16_ESM.zip › Figure 4/Mia PaCa 2 cells 30 mW 80 mW 0.003 40x obj SRS blocked 3 cropped.png]

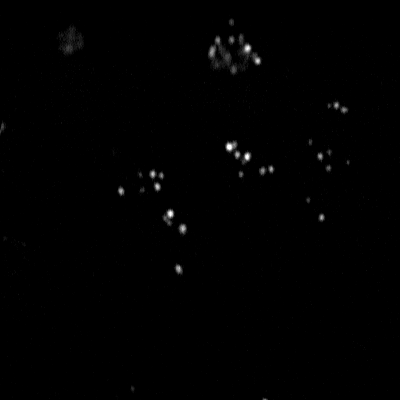

Supplement: Supplementary file 16 — Source Data [file 41467_2022_32071_MOESM16_ESM.zip › Figure 4/Mia PaCa 2 cells 30 mW 80 mW 0.003 40x obj SRS blocked 3.png]

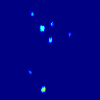

Supplement: Supplementary file 16 — Source Data [file 41467_2022_32071_MOESM16_ESM.zip › Figure 4/Mia PaCa 2 cells 30 mW 80 mW 0.003 40x obj SRS open 3 cropped 16 color.png]

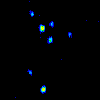

Supplement: Supplementary file 16 — Source Data [file 41467_2022_32071_MOESM16_ESM.zip › Figure 4/Mia PaCa 2 cells 30 mW 80 mW 0.003 40x obj SRS open 3 cropped 16 colot.png]

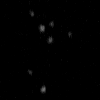

Supplement: Supplementary file 16 — Source Data [file 41467_2022_32071_MOESM16_ESM.zip › Figure 4/Mia PaCa 2 cells 30 mW 80 mW 0.003 40x obj SRS open 3 cropped.png]

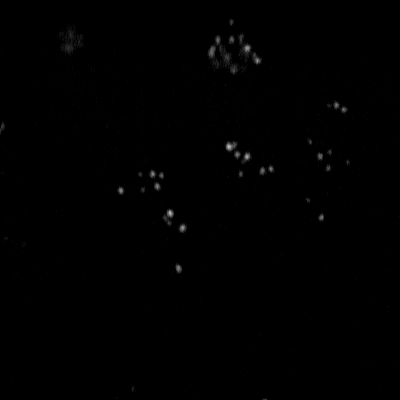

Supplement: Supplementary file 16 — Source Data [file 41467_2022_32071_MOESM16_ESM.zip › Figure 4/Mia PaCa 2 cells 30 mW 80 mW 0.003 40x obj SRS open 3.png]

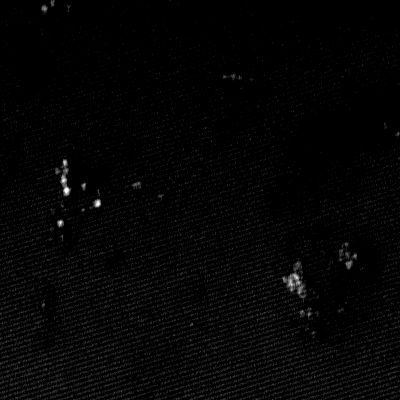

Supplement: Supplementary file 16 — Source Data [file 41467_2022_32071_MOESM16_ESM.zip › Figure 5/Mia PaCa 2 cells 30 mW 80 mW 0.003 40x ALWAYS ON open 06.png]

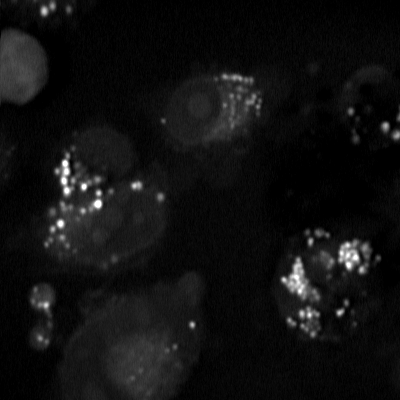

Supplement: Supplementary file 16 — Source Data [file 41467_2022_32071_MOESM16_ESM.zip › Figure 5/Mia PaCa 2 cells 30 mW 80 mW 0.003 40x CH cell.png]

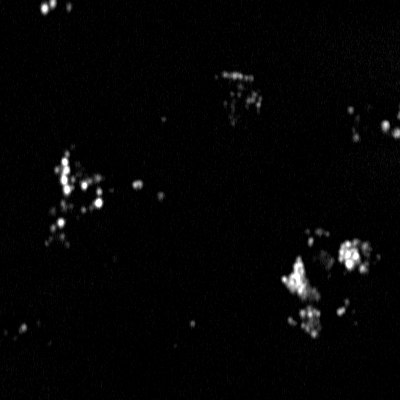

Supplement: Supplementary file 16 — Source Data [file 41467_2022_32071_MOESM16_ESM.zip › Figure 5/Mia PaCa 2 cells 30 mW 80 mW 0.003 40x CLOSED CMTE 01.png]

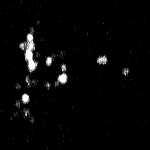

Supplement: Supplementary file 16 — Source Data [file 41467_2022_32071_MOESM16_ESM.zip › Figure 5/Mia PaCa 2 cells 30 mW 80 mW 0.003 40x two box U0.12 L0.1 active pixel 01 frame 1 #2 cropped.png]

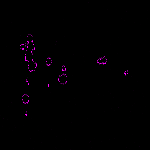

Supplement: Supplementary file 16 — Source Data [file 41467_2022_32071_MOESM16_ESM.zip › Figure 5/Mia PaCa 2 cells 30 mW 80 mW 0.003 40x two box U0.12 L0.1 active pixel 01 frame 1 Active pixels #2 cropped.png]

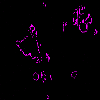

Supplement: Supplementary file 16 — Source Data [file 41467_2022_32071_MOESM16_ESM.zip › Figure 5/Mia PaCa 2 cells 30 mW 80 mW 0.003 40x two box U0.12 L0.1 active pixel 01 frame 1 Active pixels cropped.png]

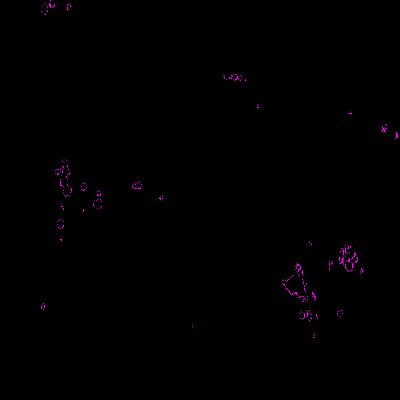

Supplement: Supplementary file 16 — Source Data [file 41467_2022_32071_MOESM16_ESM.zip › Figure 5/Mia PaCa 2 cells 30 mW 80 mW 0.003 40x two box U0.12 L0.1 active pixel 01 frame 1 Active pixels.png]

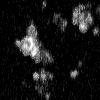

Supplement: Supplementary file 16 — Source Data [file 41467_2022_32071_MOESM16_ESM.zip › Figure 5/Mia PaCa 2 cells 30 mW 80 mW 0.003 40x two box U0.12 L0.1 active pixel 01 frame 1 cropped.png]

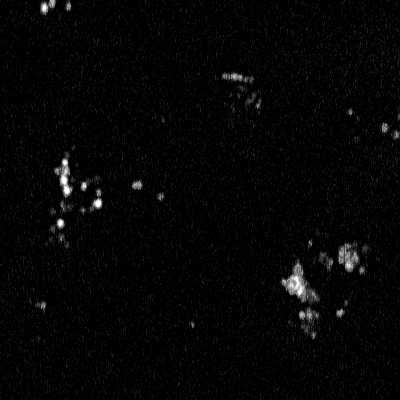

Supplement: Supplementary file 16 — Source Data [file 41467_2022_32071_MOESM16_ESM.zip › Figure 5/Mia PaCa 2 cells 30 mW 80 mW 0.003 40x two box U0.12 L0.1 active pixel 01 frame 1.png]

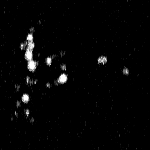

Supplement: Supplementary file 16 — Source Data [file 41467_2022_32071_MOESM16_ESM.zip › Figure 5/Mia PaCa 2 cells 30 mW 80 mW 0.003 40x two box U0.12 L0.1 active pixel 01 frame 2 #2 cropped.png]
